# Mechanical, Durability, and Environmental Impact Properties of Natural and Recycled Fiber Geopolymer with Zero Waste Approach: Alternative to Traditional Building Materials

**DOI:** 10.3390/polym17172432

**Published:** 2025-09-08

**Authors:** Haluk Görkem Alcan

**Affiliations:** Department of Civil Engineering, Kafkas University, Kars 36100, Türkiye; hgorkemalcan@kafkas.edu.tr

**Keywords:** hemp fiber, hybrid fiber, geopolymer durability, recycle, waste disposal, waste tire steel fiber

## Abstract

This study evaluates the physical, mechanical, durability, and environmental properties of geopolymer mortars (GMs) produced using waste tire steel fibers (WTSFs), hemp fibers (HFs), waste marble powder (WMP), and recycled fine aggregates (RFAs). Within the scope of this study, fibers were incorporated as single and hybrid types at 0.5% and 1% by volume. The addition of HFs generally reduced dry unit weight, as well as compressive and flexural strength but increased fracture energy by nearly three times. The addition of WTSFs improved compressive and flexural strengths by up to 42% and enhanced fracture energy by 840%. Hybrid fibers increased the strength values by 21% and the fracture energy by up to four times, demonstrating a clear synergistic effect between HFs and WTSFs in enhancing crack resistance and structural stability. In the durability tests conducted within the scope of this study, HFs burnt at 600 °C, while WTSFs showed signs of corrosion under freeze–thaw and acid conditions; however, hybrid fibers combined the benefits of both materials, resulting in an effective preservation of internal structure. The fact that the materials used in the production of GM samples were waste or recycled products reduced the total cost to 188 USD/m^3^, and thanks to these materials and the carbon-negative properties of HFs, CO_2_ emissions were reduced to 338 kg CO_2_/m^3^. The presented study demonstrates the potential of using recycled and waste materials to create sustainable building materials in the construction industry.

## 1. Introduction

Concrete is among the most preferred materials in the construction industry due to its satisfactory mechanical performance, economy, and easy access to raw materials during production [1]. The binder frequently used in concrete production is Ordinary Portland Cement (OPC). OPC has proven its criteria, such as reliability and suitability, due to evaluations made from past to present. Despite its advantages, producing one ton of cement consumes about two tons of natural resources, releases an average of 750 kg of carbon dioxide (CO_2_) into the atmosphere, and requires 3500–4000 MJ of energy [2,3]. Research has shown that cement production releases almost 1.5 billion tons of CO_2_ into the atmosphere annually and consumes 36% of global energy in the process [4,5]. These data show that the OPC production trend is not environmentally friendly, or sustainable. In the past decades, Geopolymer Concrete (GPC) has been developed as a result of efforts to produce a less energy-intensive, environmentally friendly, and more economical building material as an alternative to OPC admixed concrete. GPC is a composite material produced by activating aluminosilicate-based precursors with various alkali activators. Therefore, another name for geopolymer concrete is alkali-activated concrete (AAC) [6]. The dissolution of aluminosilicate materials with an alkaline activator is called geopolymerization. As a result of this reaction, a three-dimensional polymeric chain containing sialate bonds (Si-O-Al-O) is formed [7,8]. These bonds form the basic skeleton of the composite, determining the mechanical and durability properties of the GPC [9,10]. In the production of geopolymer concrete, mainly aluminosilicate-based industrial wastes such as ground blast furnace slag (GBFS), fly ash (FA), silica fume (SF), metakaolin (MK), and rice husk ash (RHA) are used [11,12,13,14,15]. Since the binders used in geopolymer concretes are obtained through the recycling of industrial wastes, CO_2_ emission is 80% less compared to concretes with OPC [16,17,18]. This is an outstanding advantage of GPC regarding environmental friendliness and sustainability. Other advantages of GPC compared to OPC concrete are its high mechanical strength, rapid strength gain, low shrinkage capacity, high acid and fire resistance, as well as low heat and sound conductivity [19,20,21].

Geopolymer concrete has many advantages, but its flexural strength and deformation capacity are limited due to its brittle behavior. Researchers reported that the fiber-reinforced geopolymer concrete (FRGPC) technology improved the properties of the composite, such as flexural strength, ultimate deformation capacity, and fracture toughness [22,23,24]. Due to the bridging effect, the fibers added to the GPC blend limit the formation of micro and macro cracks in the loaded composite. In this way, the flexural strength and ductility of FRGPC are increased, as well as its resistance to adverse environmental effects [25,26,27,28]. In the literature, steel or synthetic fibers such as carbon, glass, polymer, and basalt are generally used in FRGPC production [29,30]. The high tensile strength and easy commercial availability of these fibers are the main reasons they are often preferred. However, during the production of these fibers, high energy is consumed, and a lot of CO_2_ is emitted. In addition, these fibers are often expensive and difficult to recycle. For example, steel is known to be responsible for 31% of CO_2_ emissions in reinforced concrete. Also the unit cost of carbon fibers is considerably high, and the emission value is about 29.5 kg CO_2_/kg [31,32]. Using synthetic or steel fibers is not reasonable in terms of sustainability, carbon footprint, and economy. It is therefore important to utilize such industrial, agricultural, and plastic waste, and recover various materials through the recycling process [33,34,35,36]. For FRGPC to become more sustainable by reducing its carbon footprint, steel fiber from waste tires and hemp fiber, a plant waste, could offer an ideal solution.

Worldwide, 1.5 billion tires become wasted every year. This means that 17 million tons of waste tires cause environmental pollution annually. Another concern is that waste tires do not biodegrade and, in uncontrolled combustion, can form compounds harmful to human health [37,38]. Governments and local authorities have taken steps to recycle waste tires over the past decade to tackle the massive pollution caused by waste tires. Through various recycling processes, steel fibers, which are present in 8–15% of the structure of waste tires, are recovered. In studies where waste tire steel fibers (WTSFs) were used to replace industrial steel fibers, it was observed that WTSFs exhibited adequate mechanical properties and no compatibility problems [39,40,41]. The use of WTSFs in geopolymer concretes is more environmentally friendly and cheaper, and it has been estimated that using these fibers could reduce CO_2_ emissions by more than 1 billion kg per year [42]. For these reasons, using WTSFs in geopolymer applications and research should be encouraged.

Hemp is a plant that can grow in as little as 4 months with minimal water and without the need for pesticides. The hemp plant is used in many fields, such as fabric, paper, food, and pharmaceuticals [43]. Hemp fiber (HF) is a natural fiber derived from the stalk of hemp. HF has a relatively high specific strength, low cost, low density, high energy recovery, and is harmless to health [44,45]. In addition, considering the life cycle of hemp fibers, it is a carbon-negative fiber type because 1 ton of hemp can store about 1.7 tons of carbon [46,47]. The use of hemp fibers in geopolymer concretes has recently attracted interest, and it has been observed that the mechanical properties of the geopolymer composite improve with the use of HF [48,49,50]. However, studies on hemp fiber-reinforced geopolymer concrete (HFGPC) are scarce because there is no standard for this type of fiber, and its use is limited because it sometimes requires treatment. Due to its superior properties, using HF as a substitute for industrial steel and/or synthetic fibers is important regarding carbon footprint and sustainability practices.

In this study, WTSF and HF were added to the geopolymer mortar in single and hybrid forms at rates of 0.5% and 1% by volume. The effect of adding fibers of different volumes and types to the mixtures on the experimental results has been examined. In addition, since no studies were found in which these fibers were used in hybrid form, this study aims to bring a different perspective to the literature. In recent studies, waste tire steel or hemp fibers have been incorporated into geopolymer concretes. In these studies, the focus has mostly been on investigating the effects of these fibers on mechanical properties; however, various durability characteristics of HF have been neglected. Determining some durability properties of HF, a type of natural fiber, is intended to fill this gap in the literature and to provide researchers with insights into the durability behavior of organic fiber-based geopolymer mortars. In the presented study, in addition to the physical and mechanical properties of fiber-reinforced geopolymer mortar samples, durability properties such as high temperature, freeze–thaw, and acid resistance have also been examined. In this respect, this manuscript systematically investigates many properties. Just like the fibers used in this study, the binder and aggregates also consist of waste or recycled materials, which is another original aspect of this manuscript. Therefore, a comprehensive CO_2_ emission and cost analysis was carried out to evaluate the environmental and economic sustainability of the developed mortars. As a result, this study aims to produce environmentally friendly building materials, contribute to sustainable construction practices, and improve effective waste disposal.

## 2. Experimental Program

### 2.1. Materials

**Figure 1:**
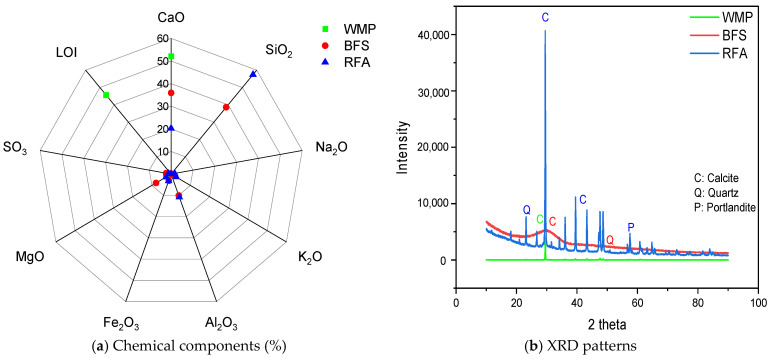
Chemical and mineralogical features of WMP, BFS, and RFA.

**Figure 2:**
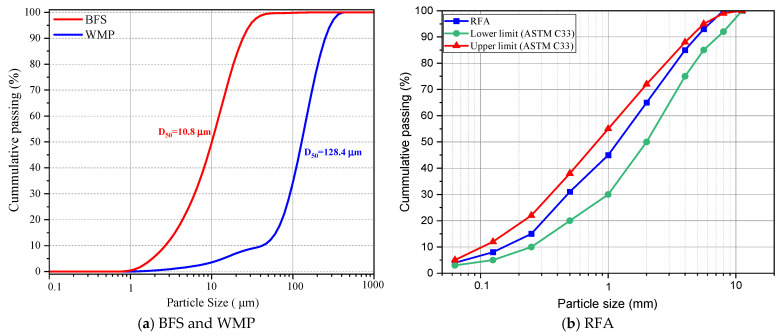
Sieve analysis of BFS, WMP, and RFA.

This study used blast furnace slag (BFS) as an alumino-silicate source to produce geopolymer mortar (GM). BFS is an industrial waste generated by iron production. Therefore, its disposal is important for sustainability. In addition, BFS has been reported to provide high mechanical and durability properties when used as a binder in geopolymer concretes [51,52]. The density of BFS is 2.88 g/cm^3^, the specific surface area (SSA) is 4700 cm^2^/g, and the average particle size (d_50_) is 10.8 µm. Figure 1a shows the chemical components of BFS and Figure 1b shows the XRD pattern of its mineralogical properties. Figure 2a presents the sieve analysis of the binder. Two types of aggregates were used in the GM samples produced within the scope of this study. One is waste marble powder (WMP), and the other is a recycled fine aggregate (RFA). In marble production, approximately 50% of marble waste is generated [53,54]. The accumulation and storage of marble wastes, which cause adverse effects on nature, are serious problems. For this reason, recycling marble waste provides industrial gains and prevents environmental problems. Worldwide, it is estimated that about 1.68 kg of construction demolition waste is generated per person per day [55]. Disposal of these wastes is quite complex and costly. Recycling and reuse of waste concrete will contribute significantly to sustainability by preventing the consumption of natural resources. The recycled aggregates used in this study were obtained by grinding the waste concrete to desired sizes. The density of the WMP and RFA used in the experiments were 2.66 and 2.65 g/cm^3^, respectively. Within the scope of this study, the total aggregate mass was designed to consist of 50% WMP and the remaining half was the RFA. The chemical components of the WMP and RFA are shown in Figure 1a, and the sieve analysis of the aggregates is presented in Figure 2a,b. The XRD patterns of both aggregates are presented in Figure 1b. In GM production, sodium hydroxide (NaOH) and sodium silicate (Na_2_SiO_3_) were used as activators. Since geopolymerization is slow when these activators are used alone, silicates and hydroxides are often used together [56,57]. The NaOH used in the experiments was 12 M, and the sodium silicate solution contained 14.2% Na_2_O, 26.8% SiO_2_, and 59.0% H_2_O by mass. The density of NaOH and Na_2_SiO_3_ are 1.61 and 1.66 g/cm3, respectively. In the experiments, the Na_2_SiO_3_/NaOH ratio was chosen as 2.5, and no additional water or superplasticizer was added.

**Figure 3:**
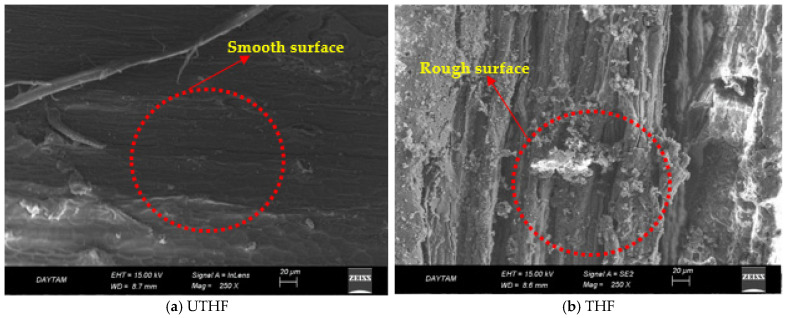
SEM images of untreated and treated hemp fibers.

The presented study used hemp fiber (HF) and waste tire steel fiber (WTSF) as mortar reinforcements. WTSFs were included in the GM mix without any pretreatment. Two types of HF were used: treated (THF) and untreated (UTHF). Since hemp fibers are of vegetable origins, they have waxy and oily structures on their surfaces, and these layers reduce fiber–mortar adherence. As a result of processing, HF is free from these layers to a certain extent and has a rougher and branched structure. This results in stronger interfacial bonding and increased ductility [58]. In this study, THF was soaked in NaOH for 24 h before being added to the mixture and then dried at 60 °C for 24 h. Since NaOH was used as an activator in the GM mixtures, using it for the treated hemp fibers was economical. UTHF was soaked in water for 24 h before being added to the GM mixture and then being subjected to the same drying process. By soaking the hemp fibers in water, hydrophilicity was reduced, and the stability of the blend was maintained [59]. Scanning Electron Microscope (SEM) images of THF and UTHF are presented in Figure 3. The length of the steel and hemp fibers was determined to be approximately 12 mm, and the fiber diameters were determined to be 0.15–0.2 mm. Therefore, the length and diameter of the fibers were not parameters in the test samples. The mechanical and physical properties of the fibers are given in Table 1. All raw materials and fibers used in the scope of this study were procured from local companies in Türkiye.

### 2.2. Mixture Proportions, Production, and Curing

**Figure 4:**
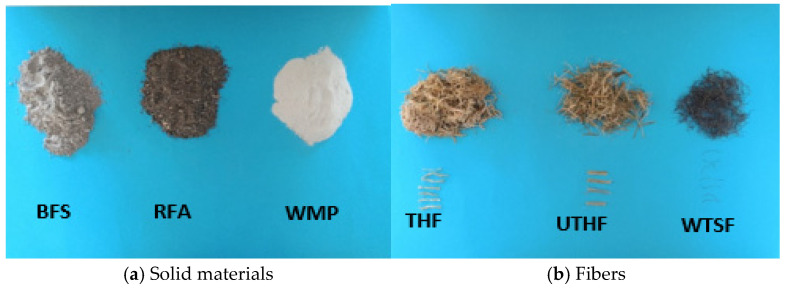
Materials used in GM production.

**Figure 5:**
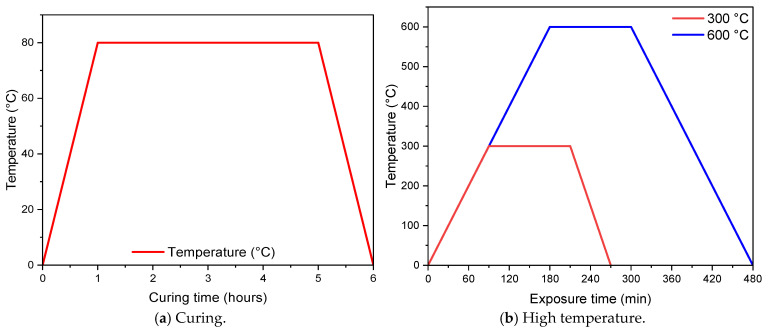
Heat procedure of samples.

Eleven mixtures were created for the experimental study. The first of these mixtures is the non-fiber reference (REF) sample, and the others have different fiber types and fiber volumes. The M1–M5 mixtures have 0.5% fiber and, the M6–M10 mixtures have 1% fiber by volume. Table 2 gives the amount by weight of all the mixture groups per 1 m^3^. According to the results of the preliminary tests, the amount of BFS was determined to be 750 kg/m^3^ in all groups, and the activator/binder ratio was 0.75. While 50% of the aggregate volume was WMP, the remaining half was the RFA. During the production of the GM mixtures, the dry materials, BFS, and aggregates were first put into the mixer and mixed for 2 min. Then, NaOH and Na_2_SiO_3_ were added to the mixer for 3 min. Finally, the fibers were added to the fresh mixture and mixed for 5 min, and the mortar was ready to pour. The concrete flow diameters of all the groups of fresh mix were measured according to the ASTM C230 [64] standard; the molds were wrapped with stretch film and placed in an oven for heat curing. It is known that GPC gains higher and earlier strength by heat curing [65,66]. According to the results of the preliminary experiments conducted for this study, all groups were subjected to heat curing at 80 °C for 4 h. For this reason, a certain additional amount of energy is required for heat curing. After curing, the specimens were removed from the molds and allowed to cool at room temperature. After cooling the specimens for 24 h, the test process was started. The materials used in the GM specimens are shown in Figure 4, and the curing process applied is shown in Figure 5.

* Fiber volume (%).

### 2.3. Testing Procedure

This study investigated the physical, mechanical, durability, and microstructural properties of WTSFs and HFs added to geopolymer mortars (GMs). All tests were carried out after heat curing. Within the scope of this study, 396 cube and 198 prism specimens were produced.

#### 2.3.1. Physical Properties

To determine the physical properties of the GM samples, dry unit weight, apparent porosity, and water absorption values were measured 24 h after casting. These tests were performed on 50 × 50 × 50 mm cube specimens following ASTM C642 [67] standards.

#### 2.3.2. Mechanical Properties

Compressive strengths, flexural strengths, and specific fracture energies were measured to determine the mechanical properties of the GM specimens. To determine the compressive strength, 50 × 50 × 50 mm cube specimens were used according to the ASTM C349 [68] standard. To determine the flexural strength, a three-point bending test was performed according to ASTM C348 [69] standards, and 40 × 40 × 160 mm prism specimens were used. Using the load-displacement data obtained during the flexural test, the specific fracture energies of the mixtures were measured according to the ASTM C1018 [70] standard. After all the durability tests (high temperature and acid effect) were applied to the GM specimens, the mentioned mechanical tests were conducted.

#### 2.3.3. High Temperature Resistance Properties

In order to measure the high temperature resistance of the GM samples, 300 and 600 °C temperatures were applied according to ASTM E119 [71] standards. A laboratory type oven was used to test high temperature resistance, and the oven’s heating was set at 10 °C/min. The heating procedure of the high temperature furnace is given in Figure 5b. For the high temperature tests, 50 × 50 × 50 mm cube and 40 × 40 × 160 mm prism specimens were used. The specimens were exposed to the specified temperature for 2 h and then cooled to room temperature. After cooling, the specimens’ compressive strengths, flexural strengths, fracture energies, and weight losses were measured.

#### 2.3.4. Freeze–Thaw Resistance Properties

According to ASTM C666-97 [72] standards, 50 × 50 × 50 mm cube and 40 × 40 × 160 mm prism GM specimens were subjected to a rapid freeze–thaw test. Two different freeze–thaw cycles, 50 and 100, were applied to the specimens. In each cycle, the samples were frozen at −20 °C for 7 h and thawed at +5 °C for 5 h. After reaching the specified number of cycles, the samples’ weight losses were measured, and mechanical tests were performed.

#### 2.3.5. Acid Resistance Properties

HNO_3_ was used in accordance with the ASTM C267 [73] standard to measure the acid resistance of the GM samples. The acid solution concentration was 5%, and the ratio of the solution to the samples was 0.9. The samples were kept in the acid solution for 90 days. The solution was renewed weekly for the first month and then every 15 days. For the acid resistance test, 50 × 50 × 50 mm cube and 40 × 40 × 160 mm prism specimens were used. Mechanical tests were performed after the GM specimens were removed from the acid solution, and weight losses were measured.

#### 2.3.6. Microstructure Properties

Microstructure images of the hemp fibers used in the experiments and of the samples were taken at different magnifications after all the experiments. ZEISS scanning electron microscope (SEM) at the Atatürk University DAYTAM unit was used for this analysis. The parts for the SEM analysis were taken from the sample center as much as possible after the mechanical tests were applied.

## 3. Results and Discussion

In this study, all the results are given in Table 3 to make the experiments’ results more understandable.

### 3.1. Physical Properties

**Figure 6:**
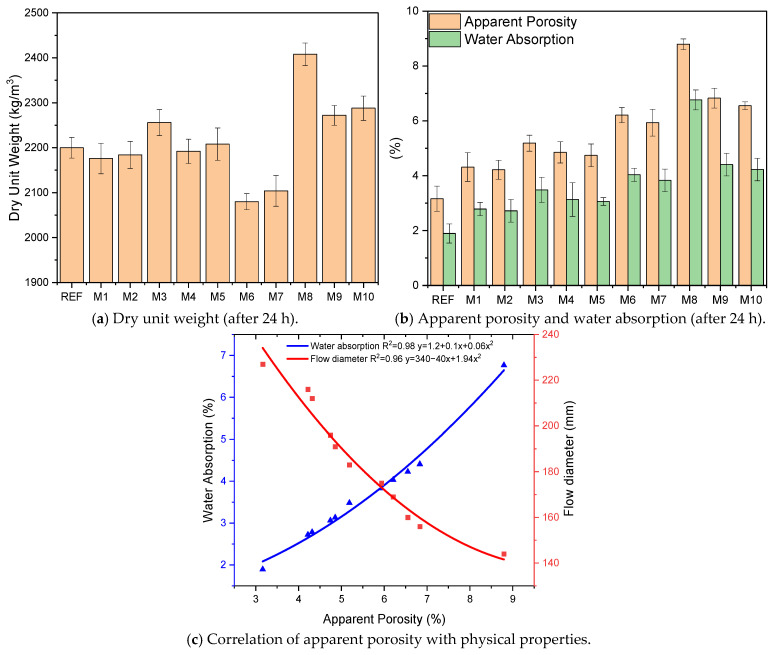
Physical properties of GM samples.

The dry unit weight results of the GM specimens are shown in Figure 6a. The dry unit weight results show that the fiber additive significantly affected the GM specimens. The non-fiber reference sample (REF) has a density of 2200 kg/m^3^ and is a benchmark for other blends. As the hemp fiber content increased, a decrease in the dry unit weight of the samples was observed. This is because hemp fibers have a lower density than mortar. M6 with 1% UTHF, for instance, had the lowest density at 2080 kg/m^3^, roughly 6% less than the reference specimen. NaOH salt is deposited on the surface of hemp fibers during treatment. Suppose the SEM images of THF and UTHF presented in Figure 3 are examined. In that case, the abundance of gray spots on the surface of THF is particularly noticeable, and these spots are the accumulated NaOH salts. Salts deposited on the surface of the fibers cause THF to be slightly denser than UTHF, as seen in Table 1. Due to this difference, the weight of the samples with treated hemp fiber additives is slightly bigger than that of the untreated hemp fiber additives. For specimens containing waste tire steel fiber (WTSF), dry unit weight increased significantly as fiber content increased. Specimen M8 (with 1% WTSF) has the highest weight of 2408 kg/m^3^, almost 10% higher than the reference specimen. This difference is due to the high density of the steel fibers (7.85 g/cm^3^). The dry unit weights of the samples with hybrid fibers are higher than those with hemp fibers only and lower than those with WTSFs only. The dry unit weight of the 0.5% hybrid fiber samples (M4 and M5) is very close to the reference, while this value is slightly higher than the reference sample in the 1% hybrid fiber samples (M9 and M10). Lv and Liu emphasized that the mixtures’ unit weight values decreased with the addition of hemp fiber to the geopolymer concretes. They stated that due to the low density of HF, the specimens became lighter [49]. Yazıcı et al. reported in their study that due to the high density of steel fibers, the unit weights of the samples increased in parallel with the increase in the steel fiber ratio used in the mixtures [74].

Figure 6b shows the GM samples’ apparent porosity and water absorption values. The apparent porosity of the REF sample was 3.16%, and the water absorption rate was 1.89%. These results are the lowest values among the GM mixtures. It was observed that the apparent porosity and water absorption values increased with the addition of fiber to the mixtures. These results reached larger values with the increase in fiber volume from 0.5% to 1%. In the samples containing hemp fibers (UTHF and THF), it was thought that the apparent porosity and water absorption rates increased due to the fibers partially absorbing the activators and forming irregular voids in the matrix. Another remarkable result is that the THF samples have lower porosity and water absorption values than those with UTHF. For example, sample M7 (1% THF) had a porosity of 5.94% and a water absorption rate of 3.83%, while M6 (1% UTHF) had 6.21% and 4.03%, respectively. This difference can be explained by the better adherence of THF to the matrix by NaOH treatment, limiting the formation of voids. When Figure 6b is examined, it is seen that the apparent porosity and water absorption values reached higher values in the samples containing WTSF. For example, the apparent porosity of M8 (1% WTSF) was 8.80%, and water absorption was 6.77%. These values stand out as the highest results among all the samples. The observation of these results is due to some reasons. The high density of WTSF restricts the flow in the mortar and the homogeneous distribution of the fibers. This prevents the mortar from enveloping the fibers homogeneously and creates irregular voids between the fiber and mortar. In addition, no plasticizers were used to produce the GM mixtures, which made them more environmentally friendly and low emission. This was another reason for the high apparent porosity and water absorption values. The apparent porosity and water absorption values of the samples with hybrid fibers (M4, M5, M9, and M10) were between the results of the samples using only HF or WTSF. This indicates that using hybrid fibers will produce more stable samples regarding porosity and water absorption than using only waste tire steel fibers. For example, M9 with 1% fiber (UTHF + WTSF), which has the highest apparent porosity and water absorption value in the hybrid groups, has 22% less porosity and 34% less water absorption than M8. Considering the apparent porosity and water absorption values of the GM samples and the flow diameters in Table 3, it is seen that there is a relationship between these results. As the porosity of the samples increased, the water absorption increased while the flow diameters decreased. This is because more voids mean more water enters the samples, while the presence of these voids restricts the flow of mortar. The R^2^ of 0.98 between apparent porosity and water absorption and the R^2^ of 0.96 between apparent porosity and flow diameter indicate this significant relationship (Figure 6c). As a result, the use of fibers in GM specimens reduces workability and increases apparent porosity and water absorption, but the presence of fibers is significant for crack control. Li et al. stated that hemp fibers carry air during mixing, so air pockets were formed in the sample. They noted that these voids can be micro in structure or larger in size due to the clustering of fibers. They realized that these air voids increased the sample’s porosity and water absorption values [75]. Öz et al. stated that fibers cause a loss of workability and increase porosity in geopolymer concretes. They emphasized that this affects a geopolymer’s fresh and hardened properties [76]. Researchers have reported that as the volume of steel fibers added to a geopolymer or concrete increases, the mixture’s viscosity decreases, resulting in increased porosity and water absorption [77,78].

### 3.2. Mechanical Properties

**Figure 7:**
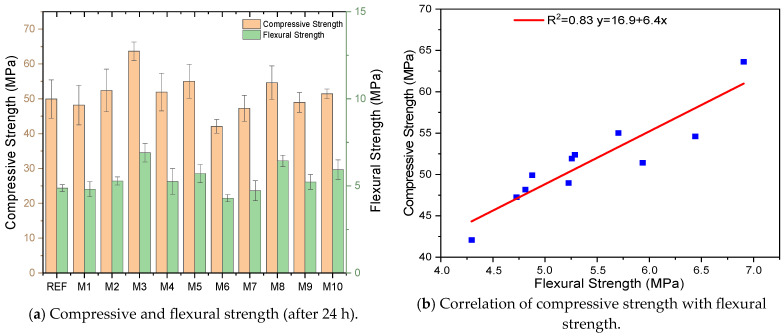
Mechanical properties of GM samples.

**Figure 8:**
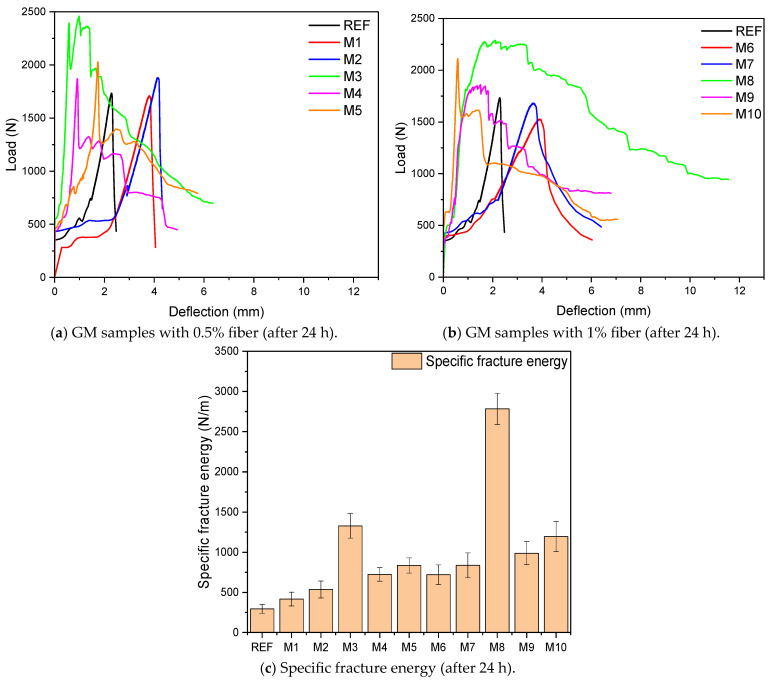
Load-deflection graphs and fracture energy of GM samples.

Figure 7 and Figure 8 present the mechanical properties of the GM specimens after 24 h, such as compressive strength, flexural strength, and specific fracture energy. When the compressive strength results are analyzed (Figure 7a), it is seen that the reference specimen’s strength is 49.9 MPa. On the other hand, the compressive strengths of the fibrous specimens showed increasing or decreasing trends. These differences directly relate to the fiber types or fiber volumes used in the GM specimens. The compressive strengths of the specimens where only hemp fibers were used (M1, M2, M6, and M7) tended to decrease compared to the reference. When the volume of hemp fibers added to the mixtures was increased from 0.5% to 1%, the compressive strength decreased more. For example, the compressive strength of specimen M6 (1% UTHF) was 42.1 MPa, the lowest value among the groups, and showed a significant decrease of 15% compared to the REF. These results are closely related to the low tensile strength of hemp fibers. Since the elastic modulus of the mortar phase is larger than that of hemp fibers, the HF admixture caused a decrease in the compressive strengths in general. Another noteworthy result here is that the THF-doped specimens showed a better performance than the UTHF-doped specimens by showing a reducing effect on strength loss. While the M6 specimen experienced a 15% compressive strength loss compared to the REF, this decrease was around 5% in the M7 specimen. This result is due to the better adherence of THF with the matrix by increasing the surface roughness, thanks to the NaOH treatment, as shown in Figure 3. The specimens with waste tire steel fiber (WTSF) generally increased the compressive strength. Unlike hemp fibers, WTSF increased the load-carrying capacity of the specimens due to its high tensile strength. M3 (0.5% WTSF) showed the highest compressive strength among the GM specimens with 63.6 MPa, an increase of 27% compared to the reference. However, the increase in WTSF volume decreased the compressive strength after a certain point. Although the strength of M8 (1% WTSF) was higher than the REF, with 54.6 MPa, it was not as high as that of M3. This result is due to the decrease in adherence between the fiber and mortar due to the deterioration of the homogeneity of the mortar when 1% WTSF is added. Figure 7a shows that the compressive strength of the hybrid fiber specimens (M4, M5, M9, and M10) tended to increase compared to the REF. This indicates that HF and WTSF exhibit a more balanced mechanical performance by restricting crack formation through the synergy effect. M5 with 0.5% fiber (THF + WTSF) showed the highest compressive strength in the hybrid group with 55 MPa, an increase of 10.2% compared to the reference. As with the specimens with only HF or WTSF, increasing the hybrid fiber volume also harmed the compressive strength values. Da Costa Santos and Archbold treated hemp fibers with NaOH and incorporated them into concrete at 0.5% and 1% volumes. They stated that HF caused capillary voids in fresh concrete during mixing; accordingly, its compressive strength decreased compared to the reference [79]. Various researchers have reported that the incorporation of steel fibers or waste tire steel fibers into geopolymer concretes increases compressive strength. They emphasized that these fibers, thanks to their high mechanical properties, are very effective in carrying the load to which the specimens are subjected and transferring it to strong sections. They also pointed out that if the fiber volume exceeds a certain ratio, the strength gain slope may decrease [80,81].

When the results are analyzed, it is observed that the changes in compressive strength are parallel to the changes in flexural strength. The high correlation (R^2^ = 0.83) between compressive and flexural strengths presented in Figure 7b supports this interpretation. The flexural strength of the specimens with only UTHF or THF was generally lower than that of the REF. The loss of strength was even greater with a 1% hemp fiber volume. Similar to the compressive strength results, M6 (1% UTHF) was the lowest group, with a flexural strength of 4.3 MPa, representing an 11% loss in strength compared to the reference. The weak tensile strength of HF and the micro voids formed by the fibers caused the loss of flexural strength. Although there were losses in the flexural strength of the specimens with HF, it can be interpreted that these losses were within an acceptable limit. Upon evaluation of the results, it is evident that the flexural strength of the THF samples exceeds that of the UTHF samples. These results were obtained by treating hemp fibers with NaOH, due to the improvement of the mechanical properties of the fibers and the increase in mortar–fiber adherence. The specimens’ flexural strengths were considerably enhanced by waste tire steel fibers (WTSFs). The specimen with the highest value, M3 (0.5% WTSF), had a strength of 6.9 MPa, 41.6% higher than the reference. This difference is mainly caused by WTSF’s high tensile strength (2570 MPa), which enables it to better distribute and carry stresses under flexural loads. Among the hybrid fiber specimens, M10 (1% THF + WTSF) has the highest flexural strength of 5.9 MPa, an increase of 21.8% compared to the reference. Specimen M5 (0.5% THF + WTSF) is very close to M10 with a flexural strength of 5.7 MPa, which is 16.8% higher than the reference. The specimens with THF had higher flexural strengths among the hybrid fiber groups. The hybrid use of HF and WTSF in the specimens increased strength by improving load distribution. Consequently, compared to the use of fiber alone, hybrid fibers showed a more balanced flexural strength behavior. Poletanovic et al. investigated the mechanical properties of NaOH-treated and untreated hemp fiber geopolymer mortars. They determined the volume of HF as 1% in their experimental studies. At the end of the study, they observed that the addition of both fiber types increased the porosity and decreased the density of the samples. Accordingly, they reported that the flexural strength of the hemp fiber-reinforced specimens was lower than that of the plain mortar [82]. Many researchers reported that adding steel fibers to geopolymers at 0.5% and 1% volumes increased flexural strength. They attributed this result to the high elastic modulus of steel fibers, which increases the tensile loads of the mixtures and prevents crack propagation [83,84].

Figure 8a,b shows the load-deflection graphs obtained from the flexural tests of the GM specimens. When the graphs are examined, the deflection values of all the fiber-reinforced groups increased compared to the reference. In other words, the fiber-reinforced groups exhibited ductile behavior compared to the REF specimen, which showed brittle fracture. This effect increases further as the fiber volume increases from 0.5% to 1%. These results are because fibers increase deflection capability by controlling crack propagation. The specific fracture energies of the GM specimens, presented in Figure 8c, were calculated by calculating the area under the load-deflection graph. The fracture energy of the reference specimen produced without fibers was 295 N/m, and its deflection capacity was 2.6 mm. These results are the lowest values among all groups. It was observed that the fracture energies of only the groups with HF (M1, M2, M6, and M7) increased by between 41% and 183% compared to the REF. Especially with a hemp fiber volume of 1%, the increase in fracture energy more than doubled. This is an indication that HF slows down crack propagation. The fracture energy of the samples containing THF was higher compared to those containing UTHF. The surface modification of the hemp fibers treated with NaOH resulted in mechanical improvement and good adherence to the mortar. In this way, THF provided a more efficient load transfer. For example, M7 (1% THF) showed an increase of 184% compared to the REF, with a fracture energy of 837 N/m. It is seen that WTSF has the most dominant effect on the specific fracture energy and deflection capacity of the GM specimens. The fracture energy of M3 (0.5% WTSF) is 1327.9 N/m, and that of M8 (1% WTSF) is 2783 N/m. In comparison to the REF, these specimens’ fracture energies rose by 350% and 842%, respectively. In terms of deflection capacity, M8 reached the highest value of 11.6 mm, which is another indication of the increase in ductility with the addition of WTSF. The high tensile strength of WTSF carried the stresses under a flexural load much more efficiently and limited the propagation of cracks with a high bridging effect. Figure 8c shows that the hybrid fiber specimens (M4, M5, M9, and M10) effectively increase the specific fracture energy. Among the hybrid groups, the M4 specimen with 0.5 fibers (UTHF+WTSF) has the lowest fracture energy, which was 145% higher than the reference. The fracture energy of the M10 sample (1% THF+WTSF) was measured as the highest value among the hybrid groups, with 1195.7 N/m. This value is 305% higher than the REF, and the deflection capacity of this group is 7.1 mm. These results indicate that the synergistic effect of HF and WTSF provided optimum fracture energy by effective adherence with mortar. Suwan et al. incorporated hemp fibers treated and untreated with different molarities of NaOH into geopolymer composites. In their study, they chose the length of the fibers to be 20 mm and the fiber volume to be 0.5% of the binder weight. They reported that both fibers increased the composites’ energy absorption and deflection capacity due to their load distributing properties. They also emphasized that the treated fibers have higher fracture energy and deflection capacity than the untreated fibers [85]. Bhutta et al. added various fibers, including steel fibers, to geopolymer mortars. They determined the fiber volumes used in the study as 0.5% and 1%. As a result of their experimental research, they observed that steel fibers increased the deflection capacity of the specimens by a large amount. They emphasized that this increase in deflection values would positively affect the fracture energy. They stated that these results were observed due to the superior bridging effect and high elastic modulus of steel fibers, limiting crack formation in mortars [86].

### 3.3. Durability Properties

In this section, the durability test results of the GM samples are presented. Within the scope of these experiments, high temperature resistance, freeze–thaw resistance, and acid resistance were examined, respectively.

#### 3.3.1. High Temperature Resistance

**Figure 9:**
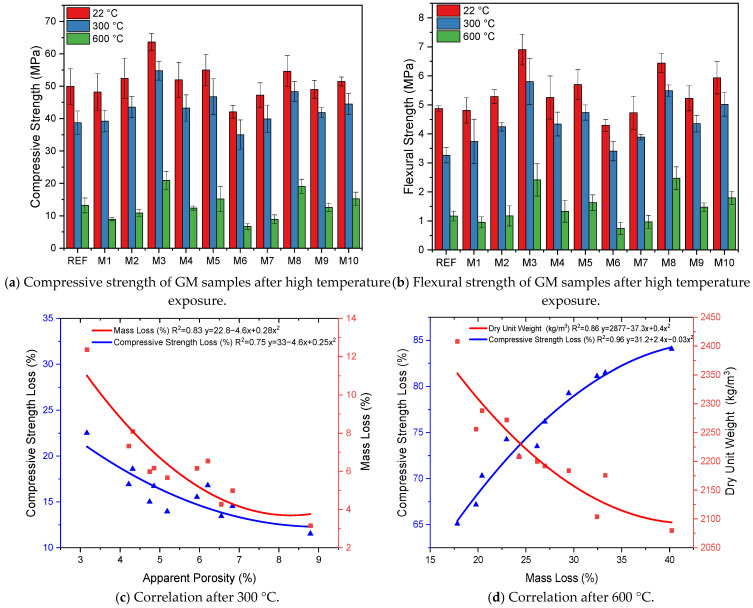
Mechanical properties and correlation of specimens after high temperature exposure.

Figure 9a,b shows the GM specimens’ compressive and flexural strength results after exposure to 300 and 600 °C. For the change in the mentioned properties of the samples to be noticeable, their values before applying high temperatures are included. The mechanical properties of geopolymer composites, such as compressive and flexural strength, remain relatively stable up to 150–300 °C. However, even at these temperatures, N-A-S-H and C-A-S-H gels slowly decompose [87,88]. The loss of water in the gel phase causes internal stresses and micro cracks, adversely affecting the mechanical properties. When the results of the samples exposed to 300 °C are examined, the compressive strength of the REF sample is 38.7 MPa, and the flexural strength is 3.27 MPa. After this high temperature, the compressive and flexural strength losses were 22% and 33%, respectively. This specimen also experienced a 12% mass loss (Table 3). These measured losses are the highest values among the GM specimens. It is observed that the addition of hemp and waste tire steel fibers to the mixtures limits the strength and mass losses in the test specimens after exposure to a high temperature of 300 °C. These results are due to the fact that the fibers reduce crack formation by limiting the internal stresses in the mortar. The specimens containing only hemp fibers (M1, M2, M6, and M7) exhibited lower strength losses than the REF. For example, specimen M7 (1% THF), after 300 °C, lost 15% compressive strength and 17% flexural strength. These results indicate that HF maintains its thermal stability at 300 °C, slowing down crack propagation due to internal stresses. As in the previous section describing the mechanical properties, the samples with THF exhibited higher strengths, i.e., less strength and mass loss, than those with UTHF. These results were because the treatment of HF with NaOH improved fiber–mortar adherence and made the fibers more mechanically stable. Only the specimens with WTSF showed lower compressive and flexural strength losses after 300 °C high temperature treatments compared to the other fiber types. The compressive and flexural strengths of specimen M8 (1% WTSF) were 48.3 and 5.5 MPa, respectively. The compressive strength loss of 11% and flexural strength loss of 14% measured in this group are the lowest values among all the GM specimens. These results are due to the high tensile strength of WTSF, which successfully reduces the internal stresses caused by the high temperature effect, and the fact that the fibers maintain their mechanical integrity at this temperature. After 300 °C high temperature treatments, the specimens containing hybrid fibers (M4, M5, M9, and M10) did not provide as high protection as those containing WTSF. However, they performed better than the specimens containing hemp fiber alone. M10 (1% THF+WTSF) lost 13% compressive and 15% flexural strength after 300 °C. At this temperature, the hybrid fibers successfully prevented the internal stresses as HF did not completely lose its adherence to the mortar, and WTSF was resistant to thermal expansion. These results show that using hybrid fibers in GM specimens provides good mechanical stability by balancing the 300 °C temperature effect. When Table 3 is examined, the losses in the specific fracture energies of the GM specimens exposed to 300 °C parallel the compressive and flexural strength losses. At this temperature value, strength decreased with the deterioration of the mortar phase, and the decrease in the deflection values caused a decrease in the fracture energies. When the samples exposed to 300 °C were visually inspected, they were found to be more gray, but there was no severe surface damage. Figure 9c shows a strong relationship between apparent porosity–mass loss (R^2^ = 0.83) and apparent porosity–compressive strength loss (R^2^ = 0.75) in the GM specimens exposed to 300 °C. At 300 °C high-temperature application, water molecules evaporating in the activator structure cause parasitic stresses in the specimens. However, if there are voids in the internal structure of the specimens, the vapor moves in these voids, thus reducing the internal stresses caused by the vapor. In the section where physical properties were explained, it was mentioned that the fibers added to the GM increase porosity. Both the presence of fibers and the increased porosity due to the presence of fibers limited the compressive strength and mass losses. Grubeša et al. added 1% HFs to a concrete mix and exposed the test specimens they produced to a 400 °C temperature. In their study, they treated hemp fibers in different ways. As a result of their experiments, they reported that hemp fibers contribute to the prevention of crack propagation at high temperatures and increase the fire resistance of concrete [89]. Lakew et al. added 0.3 and 0.6% steel fiber to fly ash/slag-based geopolymer composites. After exposing the composites they produced to 200 °C, they observed that the strength loss of the fiber-added samples was less. They also stated that the loss of strength decreased to lower levels with increasing the fiber volume. They interpreted these results to be due to the ability of fibers to inhibit crack development and propagation [90].

**Figure 10:**
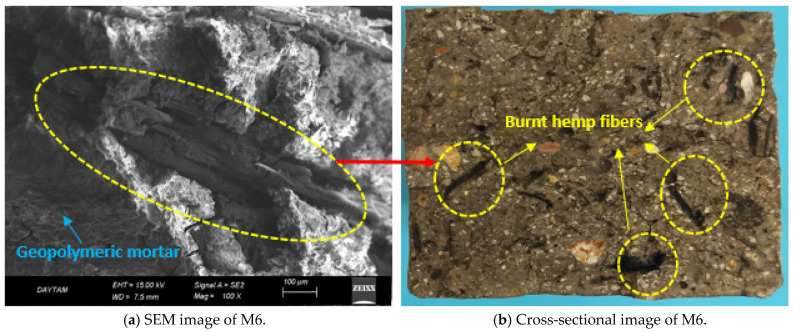
Micro and macro image of burnt hemp fibers after 600 °C.

Figure 9a,b shows the compressive and flexural strengths of the GM specimens exposed to 600 °C. When the results are analyzed, it is seen that the strength values decrease pretty dramatically. The main reason for this decrease is a significant deterioration of the gel structures that provide the strength of the specimens at 600 °C. The reference specimen exposed to 600 °C lost 73% compressive strength and 76% flexural strength. The mass loss of this specimen was approximately 27%, and the fracture energy loss was 78% (Table 3). After applying the specified temperature (600 °C), the GM specimens lost 65–84% of their compressive strength and 61–82% of their flexural strength. The most striking part of these results is that only the hemp fiber specimens (M1, M2, M6, and M7) suffered a significant loss of strength. These results are due to the complete burning of HF at 600 °C, leaving voids in the mortar. These voids negatively affected the load transfer and increased the strength loss. For example, the compressive and flexural strengths of specimen M6 (1% UTHF) were 6.7 and 0.7 MPa, respectively, the lowest values among the GM specimens. These strengths mean that the compressive strength loss was 84%, and the flexural strength loss was 82%. These results are due to the vegetable origin of hemp fibers [50]. Figure 10 presents the SEM image of the M6 sample exposed to 600 °C and the sample’s cross-sectional image after the experiment. In both images, it can be seen that the HF burned and remained as a void in the mortar. After 600 °C, the specific fracture energy of specimen M6 was measured as 28.9 (N/m) (Table 3). This value means a loss of about 96% compared to the sample not exposed to this temperature. This indicates that the fibers completely burned and lost their bridging properties. In addition, the mass loss of the M6 specimen was 40%, the most significant loss measured among the GM specimens. Even though the THF specimens performed slightly better than the UTHF specimens after exposure to the specified temperature, the strength losses were enormous. These results were due to the fact that the HFs could not withstand 600 °C, even if treated with NaOH, and burned. The conclusion from these data is that hemp fibers burn when exposed to 600 °C and have a negative effect on mechanical properties. Therefore, the high temperature exposure of composites with HF should not be ignored. When Figure 9a,b is analyzed, the highest compressive and flexural strengths among the specimens exposed to 600 °C were observed in the WTSF groups. This indicates that the steel fibers retain their thermal stability up to 600 °C. For example, the compressive strength of specimen M8 (1% WTSF) was 19.1 MPa, and the flexural strength was 2.5 MPa. These values indicate that the compressive and flexural strength losses of specimen M8 were 65% and 61%, respectively. Although WTSF retained its mechanical integrity despite exposure to 600 °C, the deterioration of the mortar phase caused the measured strength losses. In the hybrid fiber specimens, the strength losses were higher than those specimens with WTSF but lower than those with only HF. As mentioned above, the combustion of hemp fibers in the hybrid fiber groups negatively affected strength, while the presence of waste tire steel fibers enabled partial tolerance of this negativity. The losses in the compressive and flexural strengths of specimen M10 (1% THF+WTSF) after exposure to 600 °C were about 70%, supporting this interpretation. When 600 °C exposed specimens were visually inspected, the color of the specimens was light gray, and small cracks were visible on the surface. The REF was the specimen with the most intense cracks. Figure 9d shows the relationship between mass loss and compressive strength loss (R^2^ = 0.96) and mass loss and dry unit weight (R^2^ = 0.86) for the GM specimens exposed to 600 °C. The indicated high temperature application is related to the physical disintegration of the specimens, i.e., a loss of mass, and, consequently a decrease in compressive strength. In addition, Figure 9d shows that specimens with a higher dry unit weight, i.e., specimens with a higher WTSF volume, have less mass loss. Bayraktar et al. incorporated HF into BFS-based geopolymer composites. They determined the lengths of HFs as 10 and 20 mm and the fiber volume as 0.5, 1, 2, and 3% of the binder. They exposed the specimen they produced to 250, 500, and 750 °C. As a result of their experiments, they interpreted that there was no loss in the strength of the specimens exposed to 250 °C and that this result was due to the thermal stability of HF at this temperature. They reported that as the temperature increased to 500 and 750 °C, the strength and mass losses in the samples increased due to the combustion of cellulose in the HF structure [91]. Doğruyol et al. included 0.4 and 0.8% by volume of WTSF in concrete. They exposed the specimens they produced to 400, 600, and 800 °C. They reported that the WTSF specimens experienced less strength and mass loss at increasing temperatures than the reference specimens. Thanks to the properties of WTSFs, such as their ability not to degrade at applied temperatures and their high elastic modulus, they reported that WTSFs limit crack formation by meeting the internal stresses in the specimens [92].

#### 3.3.2. Freeze–Thaw (F-T) Resistance

**Figure 11:**
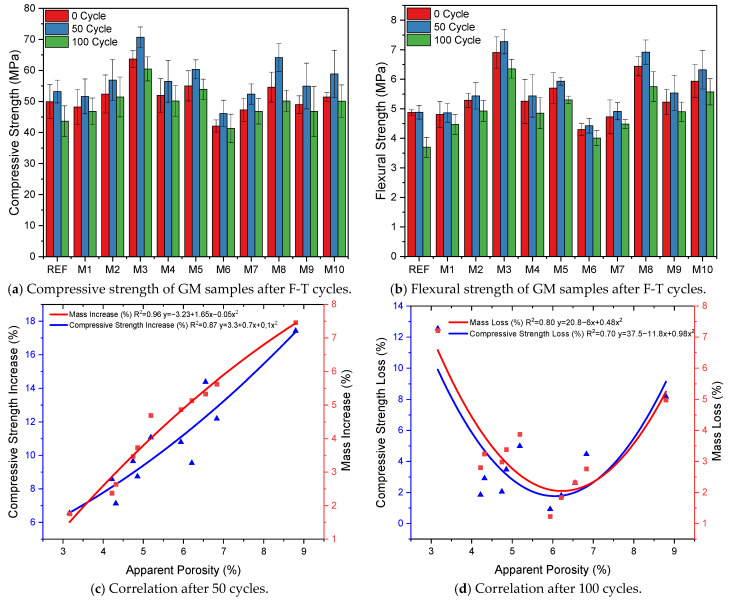
Mechanical properties and correlation of specimens after F-T cycles.

Figure 11a,b shows the compressive and flexural strengths of the GM specimens after freeze–thaw (F-T) cycles. When the results are analyzed, it is observed that the strength values of the GM specimens increase after 50 cycles. The F-T test is based on the specimens being exposed to low temperatures (−20 °C) at a certain wetness and then rising above +4 °C. This cycling causes a loss of strength as it degrades the physical and mechanical structure of the composite [93]. However, it is noteworthy that the opposite was observed in the GM specimens after 50 cycles. For example, after 50 F-T cycles, the compressive and flexural strengths of the REF specimen were 53.2 and 4.9 MPa, respectively. These results indicate that the compressive strength increased by 6% and the flexural strength increased by 0.2% compared to the specimens not subjected to freeze–thaw cycles. Adding fiber to the GM specimens increased their compressive strengths by between 7 and 17%, and flexural strength increased by between 1 and 7% after 50 F-T cycles. In particular, the increase in the strength of specimens with 1% fiber volume was higher than that of the specimens with 0.5%. The compressive strength of specimen M8 (1% WTSF) after 50 F-T cycles increased by 17%, with a strength of 64.1 MPa, and the flexural strength increased by about 7%, with a strength of 6.9 MPa. According to these results, M8 is the group with the highest strength increase after 50 F-T cycles among the GM specimens. Only the hemp fiber specimens (M1, M2, M6, and M7) showed increased compressive and flexural strengths after 50 cycles. In this group, the increase in the strength of the specimens with 1% fiber volume was higher than those with 0.5%. Another remarkable result is that the THF specimens gained more compressive and flexural strengths than the UTHF specimens. It is seen that the compressive and flexural strengths of the hybrid fiber specimens (M4, M5, M9, and M10) increased after 50 cycles, as in the other groups. For example, the compressive strength of specimen M10 (1% THF+WTSF) was 58.8 MPa, and its flexural strength was 6.3 MPa. According to these results, its compressive and flexural strengths increased by 14% and 6%, respectively. As can be seen, all the GM specimens’ compressive and flexural strengths increased after 50 F-T cycles. The increase in these values not only increased the specific fracture energies of the specimens, as seen in Table 3, but also caused the specimens to have a positive mass change, that is, an increase in unit weight. The improvement of the mechanical properties of the GM specimens after 50 F-T cycles is mainly related to the void structure in the mortar structure. During the test, water molecules diffuse into the voids in the internal structure of the specimens. The water in the voids makes the mortar more compact and increases its freeze–thaw resistance, it also hydrates with the BSF particles that have not reacted with activators. As a result of this reaction, CSH gel is formed, which contributes positively to the strength of the specimens. It was mentioned in the previous section that the water absorption values increase with an increasing apparent porosity of the specimens. This is the reason for the increases in strength and mass in the specimens with a high void ratio. This is evidenced by the R^2^ values of 0.87 and 0.96 between the apparent porosity–compressive strength increase and the apparent porosity–mass increase, respectively, as shown in Figure 11c. Aygörmez et al. investigated the freeze–thaw resistance of metakaolin-based and 0.8% polypropylene fiber geopolymer composites after 56 cycles. As a result of their experimental study, they observed that the mechanical properties of the samples increased after 56 cycles. They emphasized that this increase is due to the continued formation of geopolymeric products as moisture fills the voids of the samples [94]. In their study, Farhan et al. incorporated different volumes (1.25 and 2.5%) and lengths (6 and 12 mm) of steel fibers into BFS-based geopolymer composites. After subjecting the specimens to 90 F-T cycles, they observed increases in compressive strength. They stated this increase was much higher in the specimens with a 2.5% volume of 6 mm fibers. Their study interpreted the increase in compressive strength after F-T cycles as the continuation of the reaction of BFS due to moisture/humidity conditions [95].

**Figure 12:**
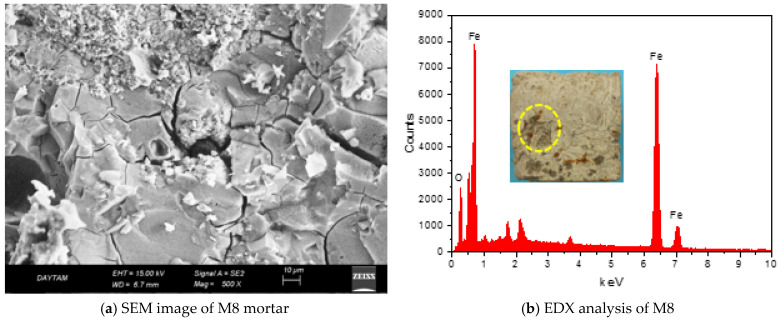
SEM image and EDX analysis after 100 F-T.

Figure 11a,b shows that the GM specimens’ compressive and flexural strengths after 100 cycles decreased differently from how they did after 50 cycles. After 100 F-T cycles, the compressive and flexural strengths of the REF specimen decreased by 12 and 24%, respectively. These results are the largest strength losses measured among the GM specimens. If the results are analyzed, the strength losses of the specimens are limited by the fiber effect. The compressive strength losses of the specimens with fibers were between 1% and 8%, and flexural strength losses were between 5% and 10%. As is known, water expands by about 9% during freezing, and this expansion causes internal stresses in the water-filled pores in the matrix. These stresses cause cracks in the specimen [96]. If the F-T cycle continues, the cracks coalesce and reach larger sizes. In this process, fibers physically and mechanically support the mortar phase, limiting the formation of micro cracks. When the results are analyzed, it is seen that only the specimens containing WTSF (M3 and M8) have more compressive and flexural strength losses compared to the other groups. The compressive and flexural strengths of specimen M8 (1% WTSF) were 50.2 MPa and 5.7 MPa, respectively. These values indicate that M8 lost 8% and 10% of its compressive and flexural strengths, respectively. The main reason for this result is the corrosion of steel fibers. Corrosion occurs when metals interact with oxygen, water, or other chemicals. As a result of these reactions, an oxide layer forms on the surface of the metal. This layer reduces the mechanical strength of the metal and causes a decrease in the adherence between the mortar and fiber [97]. With the increase in fiber volume, the strength loss of M8 was higher than M3 due to the increase in the amount of corroded fibers. As a result, the waste tire steel fibers started to corrode after 100 F-T cycles. The oxide layer formed on the surface of the fibers reduced mortar–fiber adhesion and impaired the volumetric stability. Although the specimens with WTSF provided freeze–thaw resistance compared to the REF, they lost more strength than the other groups. Figure 12 shows the SEM image of the M8 specimen mortar after 100 F-T cycles and an EDX analysis of the fiber on the surface. The SEM image shows the propagation of micro cracks in the mortar phase due to internal stresses caused by the F-T cycle. As a result of the EDX analysis, the ‘O’ (oxygen) peaks indicate corrosion in the fibers. When Figure 11a,b is analyzed, the strengths of only the specimens with HF (M1, M2, M6, and M7) after 100 cycles are quite remarkable. The compressive strength losses observed in these specimens were at most 2%, and the flexural strength losses were 5–7%. These results indicate that hemp fibers exhibit superior performance against freeze–thaw cycles. For example, M7 (1% THF) lost 0.93% in compressive strength and 5% in flexural strength. These results are the lowest values measured among all the GM specimens. In addition, as can be seen from Table 3, the mass loss of specimen M7 is approximately 1%, and its fracture energy loss is 8%, which are the lowest values. These results are related to the cellulosic wall and Ca in the structure of plant-based HF [98]. These properties of hemp fibers provide them with natural freeze–thaw resistance. In this way, HF successfully limited the internal stresses of the mortar phase while maintaining the integrity of the specimens after the F-T cycles. According to the results obtained, THF has slightly superior freeze–thaw resistance compared to UTHF. This is related to the fact that treatment with NaOH improves hemp fiber’s adherence and mechanical properties. After 100 cycles, it is seen that the strength losses of the specimens with hybrid fibers are more limited than those with WTSF only. As a result, the compressive and flexural strengths of M10 (1% THF+WTSF) and M8 (1% WTSF) are very close. While M10 experienced about a 2% loss of compressive strength and a 6% loss of flexural strength, these losses were much higher in M8. It was observed that the presence of HF in the hybrid fiber specimens offset the corrosion-induced losses of WTSF. Thus, even after 100 cycles, the hybrid fiber specimens had high compressive and flexural strengths. When the specimens exposed to 100 F-T cycles were visually inspected, map cracks were observed on the surfaces of the specimens. Small corrosion marks were observed on the surfaces of the WTSF specimens, especially in the areas of the steel fibers. Figure 11d shows the relationship between apparent porosity and compressive and mass losses. The relationship between the apparent porosity and compressive strength loss (R^2^ = 0.7) shows that the strength loss decreases with increasing porosity. This is due to the limitation of strength loss of the GM specimens caused by the presence of HF and hybrid fibers. However, after the porosity reaches a certain level, the compressive strength losses start to increase. The reason for this increase is the increase in strength loss due to the corrosion of WTSFs. Since mass loss and strength loss are interdependent, the relationship between apparent porosity and mass loss (R^2^ = 0.8) is high. Ghosn et al. incorporated hemp fibers of different lengths and different shapes into concrete at a rate of 0.75% and subjected the specimens to 144 freeze–thaw cycles. As a result of their experiments, they stated that the hemp fiber specimens showed superior freeze–thaw resistance compared to plain concrete. They reported that this result is due to the ability of hemp fibers to limit crack formation without degradation at low temperatures [99]. Mohebi et al. reported that geopolymer composites provide better F-T resistance as the unit weight decreases [100]. Various researchers have reported that the corrosion of steel fibers occurs depending on the number of freeze–thaw cycles. They found that the corrosion mechanism accelerates, especially when the crack width exceeds 0.25 mm. In addition, they emphasized that corrosion after F-T cycles negatively affects the mechanical and durability properties of the composite [101,102].

#### 3.3.3. Acid Resistance

**Figure 13:**
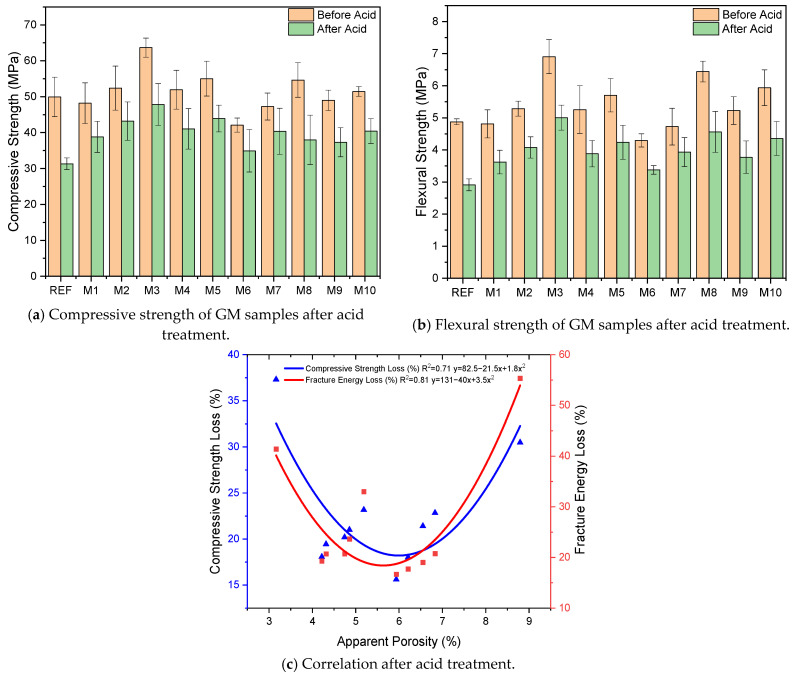
Mechanical properties and correlation of specimens after acid treatment.

**Figure 14:**
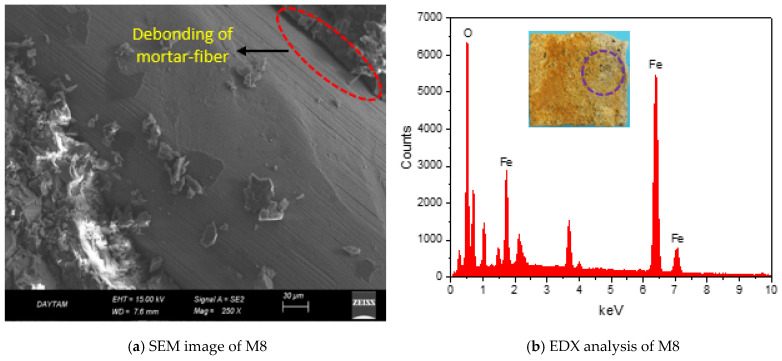
SEM image and EDX analysis after acid treatment.

Figure 13a,b shows the compressive and flexural strengths of the GM specimens before and after HNO_3_ treatment. The results show that 90 days of acid solution treatment significantly decreased the strength of the specimens. After the acid treatment, the REF specimen’s compressive strength and flexural strength were measured as 31.3 MPa and 2.9 MPa, respectively. These results indicate a 37% and 40% loss in strength, respectively. It is known that acids destroy Si-O-Al bonds in geopolymer composites, causing a void internal structure [103]. The strength loss observed in the specimen is mainly due to the breakdown of geopolymeric gel structures. In the fibrous specimens, it is seen that the strength losses are at lower levels compared to the reference. In general, the compressive strength losses in the fibrous specimens are between 17% and 30%, and the flexural strength losses are between 16% and 29%. These results indicate that the fibers added to the GM specimens restrict the formation of voids and make the specimens resistant to the acid effect. The resistance of the samples against the acid effect showed differences according to fiber type and fiber volume. Notably, the strength losses seen only in the specimens with WTSF (M3, M8) after the acid effect are significant. The losses of compressive and flexural strengths in specimen M8 were approximately 30%, while in specimen M3, these strength losses were between 24% and 27%. Just as in the freeze–thaw test, corrosion of the WTSFs by acid action is the main reason for these results. The aggressive acidic environment formed a dense oxide layer on the WTSF. The yellow color on the specimen face, seen in Figure 14b, can give an idea about the oxide density. The corrosion of WTSF weakens the adherence with the mortar and the mechanical properties of the fiber. Under the influence of acid, the strength loss of the mortar phase and the corrosion of the WTSF combined to increase the strength loss. Since the dense corrosion layer formed in the WTSF also limits the crack control capacity of the fibers, losses in specific fracture energy occur, as shown in Table 3. Figure 14 shows the SEM image and EDX analysis of specimen M8. The SEM image (Figure 14a) shows the debonding between the mortar and fibers due to the corrosion layer. Debonding has caused the fibers to lose their bridging capability and has led to a loss of strength. The ‘O’ peak in the EDX analysis taken from the WTSF on the specimen surface indicates that there is a dense corrosion layer in the fiber.

When the compressive and flexural strength results are examined, it is seen that only the hemp fiber specimens (M1, M2, M6, and M7) are the specimens with the least strength loss due to the acid effect. This group realized 14–19% compressive losses and 16–24% flexural strength losses. The lowest strength loss among the GM specimens was observed in the M7 (1% THF) specimen. These results show that hemp fibers’ acid resistance is better than that of waste tire steel fibers. The lignin, hemicellulose, and cellulose layers in the structure of HF make the fiber resistant to acid. In some studies, low dilutions of acid solutions are even used to treat hemp fibers [61,104]. With the removal of waxy and pectin structures from acid-treated HFs, the physical and mechanical properties of the fiber improve. As observed from the present study, HF retained its structural integrity after acid treatment and successfully tolerated the loss of strength in the mortar phase. It was observed that the strength losses measured in the THF specimens were slightly less than those in the UTHF specimens. This is due to the better adhesion of the treated hemp fibers to the mortar and better absorption of acid-induced internal structural stresses. When the results are examined, it is seen that the acid resistance of the specimens with hybrid fibers is higher than that of those with only WTSF. For example, the compressive strength of M10 (1% TSH+WTSF) is 40.4 MPa, which is higher than M8 with a strength of 38 MPa. The synergistic effect of hybrid fibers has a positive effect on the mechanical properties of the specimens, and the disadvantages of one fiber can be tolerated by the other. This limits the micro cracks caused by acid action and provides more balanced protection against the chemical dissolution of the mortar phase. A visual inspection of the acid-exposed specimens showed corrosion on the surfaces of the specimens and some minor defects at the corners. The WTSF specimens had noticeable corrosion marks on their surfaces. Figure 13c shows the relationship between porosity and fracture energy and the compressive strength losses of the GM specimens after acid exposure. As can be seen from the graph, increasing porosity first showed a decreasing effect on both compressive and fracture energy losses. This is an indication that the addition of fiber makes the specimens resistant to acid. Therefore, a relationship exists between porosity–fracture energy loss and porosity–compressive strength loss, with R^2^ = 0.81 and R^2^ = 0.71, respectively. The loss of fracture energy and compressive strength starts to increase as the porosity exceeds a certain point due to the negative effect of WTSF on adherence and strength due to corrosion. Saranya et al. incorporated 0.25, 0.5, and 0.75 volumes of steel fibers into BFS-based geopolymer concretes. After exposing the specimens to acid for 180 days, they measured the mass and strength losses of the specimens. According to their experimental study results, the specimens with steel fibers showed better acid resistance than the reference. They also reported that there was no improvement in mass and strength with an increasing fiber volume [105]. Thanushan and Sathiparan investigated the acid resistance of banana and coconut fiber-reinforced cement-stabilized soil blocks. These fibers are very similar in composition to hemp fiber. The researchers included both fibers in the mixture after treating them with NaOH. After the acid effect, they observed that both fibers experienced less strength loss compared to the reference. They stated that the fact that the vegetable fibers used were not affected by acid made the samples acid resistant [106].

### 3.4. CO_2_ Emission and Cost Analysis of Samples

**Figure 15:**
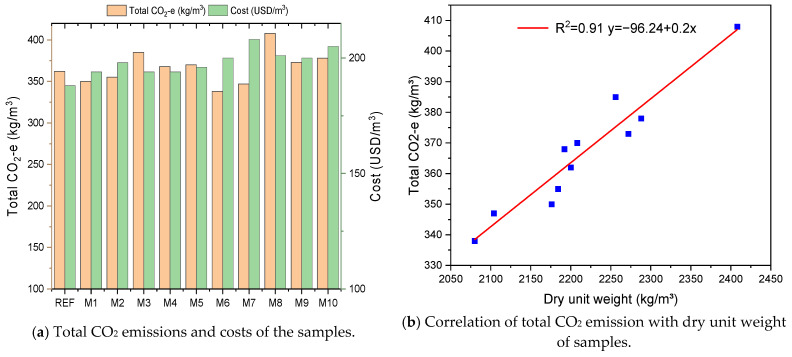
Emission analysis and correlation of total CO_2_ emission with dry unit weight of GM samples.

The total CO_2_ emission (CO_2_-e) value and total cost (USD/m^3^) of the GM samples are presented in Figure 15a. The total CO_2_-e and cost values of the samples were calculated based on the emission value (kg CO_2_/kg) and unit price (USD/kg) of the materials given in Table 4. The emission and cost values for each material were calculated by taking into account not only physical processes such as grinding, filtration, and pyrolysis but also transportation values. In addition, within the scope of the presented study, CO_2_-e and cost analyses resulting from heat curing for each group were also included in the calculation. When analyzing the CO_2_-e values and costs resulting from heat curing, the data presented in various studies were utilized, and using Formulas (1) and (2), these were included in the total emission and cost values [107,108]. If the results are analyzed, the CO_2_ emission of the REF sample was calculated as 362.9 kg/m^3^. The graph shows that M6 (1% UTHF) has the lowest CO_2_ emission among the GM samples, with a value of 338.7 kg/m^3^. This is due to the carbon-negative property of untreated hemp fiber (UTHF). It can be seen that the samples with THF have a slightly higher CO_2_-e value compared to those with UTHF. This is due to the treatment of HF with NaOH, which increases the unit kg CO_2_/kg value. It is seen that the emission values of the samples increase with the addition of WTSF to the GM mortar. For example, M8 (1.0% WTSF) has the highest CO_2_ emission value among the GM samples with 408.7 kg/m^3^. This result is due to WTSF’s relatively high carbon footprint. The hybrid fiber specimens exhibited balanced behavior, with a CO_2_-e value comparable to those of the specimens using only HF or WTSF. Taking Formula 1 into account, the CO_2_-e value resulting from heat curing has been calculated as approximately 97 kg/m^3^ for each sample. It should also be noted that heat curing increases the total emission values of GM samples. In studies conducted by researchers, it has been stated that the CO_2_ emission value of Ordinary Portland Cement (OPC) is around 400 kg CO_2_/m^3^ [109]. With an increase in cement dosage, plasticizers, and fibers such as carbon to a concrete mix, the CO_2_-e value can be more than 1000 kg CO_2_/m^3^ [110]. Considering the range of values OPC has, the GM specimens had CO_2_-e values of 338–408 kg CO_2_/m^3^, which are relatively low. These results reveal that GM samples with very low CO_2_ emissions are produced without compromising their strength properties and contribute to the development of sustainable building materials.

Unit costs were received from suppliers on 27 July 2025.

CO_2_ (kg/m^3^) = E × EF (1)

USD/m^3^ =E × EUP (2)

where E is electricity consumption (kWh/m^3^), EF is the grid emission factor (kg CO_2_/kWh), and EUP is the electricity unit prize (USD/kWh).

When the total cost results in Figure 15a were analyzed, the REF specimen had the lowest cost among the GM specimens, with a value of 188 USD/m^3^. The costs of the fibrous specimens increased depending on the fiber type and fiber volume used. The striking result here is that the cost increase in the samples with HF is slightly higher than those with WTSF. This is because hemp fibers’ unit cost is higher than that of waste tire steel fibers. In regions where hemp fibers are easier to produce and access, the unit cost of these fibers may be lower. The unit cost of THF is higher than that of UTHF, with a cost of 1.12 USD/kg, because the amount of NaOH consumed to treat the fibers is also considered when calculating the unit cost of THF. Therefore, sample M7 (1% THF) has the highest cost among the GM samples, at 208.9 USD/m^3^. Due to the increasing importance of recycling waste tires, the number of recycling facilities has increased locally in many regions. Thus, the unit cost of WTSF is decreasing day by day. M8 (1% WTSF) costs 201.2 USD/m^3^, 7% more than the REF and 4% less than M7. If the total cost of the hybrid fiber specimens is examined, it is seen that the cost of the specimens using only HF or WTSF is between the costs of the other specimens. These results show that the GM samples with hybrid fibers are optimum in terms of cost. Taking Formula (2) into account, the cost value resulting from heat curing has been calculated as approximately 23 USD/m^3^ for each sample. It is observed that heat curing also slightly increases the total cost values of the GM samples. According to the 2023 unit prices, the total cost of conventional concrete (OPC) is 215 USD/m^3^ on average, and it is emphasized that the cost may increase with the addition of fiber to concrete [117]. Considering this value, it is understood that the GM samples produced have low costs.

Figure 15b shows a positive correlation (R^2^ = 0.91) between the total CO_2_ emissions of the GM samples and their dry unit weights. That is, as the dry unit weight of the mixture increased, the total CO_2_-e value increased. This relationship is directly related to the fiber type and fiber volume added to the mortar phase. Increasing the volume of hemp fibers decreased the unit weight of the GM samples and significantly reduced the emission values. This is a noticeable effect of the carbon-negative property of HF. Although increasing the volume of WTSF increased CO_2_-e values, they are still significantly lower compared to OPC. Furthermore, although WTSF has higher emission values among the GM samples, it is a sustainable option for waste management and reduced consumption of natural resources. The balanced behavior of the hybrid fiber specimens, both in terms of mechanical and durability properties and CO_2_ emission values, is a result of the synergetic effect of the fibers in these specimens. Alcan et al. incorporated different volumes of carbon and glass fibers into geopolymer composites in a single or hybrid form. Their experimental study found that the CO2-e values of the samples ranged from 342 to 844. They reported that the carbon fibers added to the mixtures significantly increased the emission values. They also reported that the total cost of the samples was between 187 and 384 USD/m^3^, depending on the fiber type and volume. [118]. In their study, Nikbin et al. included steel fibers at 0.25, 0.5, and 1% in a concrete mix. As a result of various experiments, they observed that the mechanical properties improved significantly with a 1% fiber volume. However, they observed an increase in the CO_2_ emissions of the specimens with an increasing steel fiber volume. They stated that the CO_2_-e value of the specimen with 1% steel fiber was 652 kg CO_2_/m^3^ and emphasized that this was due to the high emission value of industrial steel fibers [119].

## 4. Limitations and Practical Implications

The heat curing (80 °C) performed in this study may directly limit field applications. The degradation of hemp fibers (HFs) at ≥600 °C restricts high temperature applications. Small samples may provide higher homogeneity; therefore, scaling studies are necessary.

## 5. Conclusions

The use of waste and recycled materials in various fields is essential for sustainability. In this study, industrial waste blast furnace slag (BFS) and waste marble powder (WMP), as well as a recycled fine aggregate (RFA), were used for the production of geopolymer mortars. In addition, hemp fiber (HF) and waste tire steel fiber (WTSF), an organic waste, were added to the mortar mixtures at 0.5% and 1% by volume, singly or in combination. The general results of various experiments performed on these environmentally friendly specimens are presented below.

⮚ Waste and recycled materials (BFS, WMP, RFA, HF, and WTSF) can be effectively utilized in geopolymer mortars, providing both performance and sustainability benefits.
⮚ As the volume of WTSF in the blend increased, the dry unit weight of the samples increased, while the opposite was observed with HF, where the weight decreased with an increasing HF volume. The dry unit weight measured in the hybrid use of fibers was between those measured in the single-use blends.
⮚ The flow diameter decreased with the addition of fiber to the samples. As the fiber volume increased, the flow diameter reached lower values. With the decrease in the flow diameter, the apparent porosity and water absorption values increased because the mortar could not cover the fibers sufficiently.
⮚ The addition of WTSF significantly enhanced compressive and flexural strengths, while HF reduced these strengths but markedly increased fracture energy. The hybrid fibers balanced these effects, achieving stable mechanical performance and demonstrating a synergistic action in improving crack resistance.
⮚ Durability assessments showed that the hybrid fibers contributed to superior structural stability: HF degraded at 600 °C and WTSF corroded under freeze–thaw cycles and acid attack, but using hybrid fibers mitigated the overall performance losses.
⮚ The integration of waste-based constituents substantially lowered environmental impact, with CO_2_ emissions reduced to ~338 kg/m^3^ and production costs reduced to ~188 USD/m^3^, underlining the economic and sustainabilty feasibility of these mixtures.
⮚ In general, this study emphasizes that geopolymers mortars incorporating WTSF, HF, and the hybrid use of both fibers not only enhance mechanical and durability performance but also support sustainable construction practices.

## Figures and Tables

**Table 1:** Properties of the fibers.

Fiber Type	Fiber Length (mm)	Density (g/cm^3^)	Tensile Strength (MPa)
WTSF	12	7.85	2570
THF	12	1.8	600–1070 [60,61]
UTHF	12	1.4	270–900 [62,63]

**Table 2:** Material quantities of mixtures (kg/m^3^).

	BFS	NaOH	Na_2_SiO_3_	WMP	RFA	UTHF	THF	WTSF	* V_f_
REF						-	-	-	0
M1						7	-	-	0.5
M2						-	9	-	0.5
M3						-	-	39	0.5
M4						3.5	-	19.5	0.5
M5	750	161	402	429	428	-	4.5	19.5	0.5
M6						14	-	-	1
M7						-	18	-	1
M8						-	-	78	1
M9						7	-	39	1
M10						-	9	39	1

* Fiber volume (%).

**Table 3:** All tests results of GM specimens.

		REF	M1	M2	M3	M4	M5	M6	M7	M8	M9	M10
	Flow diameter (mm)	227	212	216	183	191	196	169	175	144	156	160
After 24 h	Com. strength * (MPa)	49.9	48.2	52.4	63.6	51.9	55	42.1	47.3	54.6	48.9	51.4
	Flexural strength (MPa)	4.9	4.8	5.3	6.9	5.3	5.7	4.3	4.7	6.4	5.2	5.9
	Fracture energy (N/m)	295	416	535.3	1327.9	723.5	834.2	719.2	837	2783	988.2	1195.7
	Max. def. ** (mm)	2.6	4	4.8	6.3	4.9	5.7	6	6.4	11.6	6.8	7.1
	DUW *** (kg/m^3^)	2200	2176	2185	2259	2193	2210	2080	2106	2408	2273	2291
After 300 °C	Com. strength * (MPa)	38.7	39.2	43.5	54.8	43.3	46.8	35	39.9	48.3	41.9	44.5
	Flexural strength (MPa)	3.3	3.7	4.2	5.8	4.3	4.7	3.4	3.9	5.5	4.4	5
	Fracture energy (N/m)	218.1	348.2	466.1	1211.7	651.5	756	632.3	742.4	2588.4	897	1097.9
	Max. def. ** (mm)	2	3.2	4	5.7	4.1	4.4	4.6	5.8	9.9	5.9	6
	DUW *** (kg/m^3^)	1931	2004	2029	2131	2057	2076	1945	1975	2332	2158	2190
After 600 °C	Com. strength * (MPa)	13.23	8.92	10.87	20.90	12.37	15.20	6.71	8.92	19.07	12.61	15.28
	Flexural strength (MPa)	1.2	0.9	1.2	2.4	1.3	1.6	0.7	0.9	2.5	1.5	1.8
	Fracture energy (N/m)	64.8	42.9	53.9	412.6	128.3	170.8	28.9	40.7	1116.1	179.9	255.1
	Max. def. ** (mm)	1.3	1	1.2	3.2	2.1	2.6	0.9	1.1	5	2.9	3.2
	DUW *** (kg/m^3^)	1608	1452	1540	1809	1601	1672	1243	1422	1979	1750	1821
After 50 F-T	Com. strength * (MPa)	53.2	51.6	56.9	70.7	56.5	60.3	46.1	52.4	64.1	54.9	58.8
	Flexural strength (MPa)	4.89	4.87	5.44	7.28	5.44	5.94	4.43	4.92	6.92	5.54	6.32
	Fracture energy (N/m)	307.1	458.2	596.4	1585.3	823.1	958.2	798.5	946.7	3565.1	1167.7	1428.6
	Max. def. ** (mm)	2.9	4.5	5.3	7.2	5.4	6.3	6.6	7.1	12.9	7.5	7.8
	DUW *** (kg/m^3^)	2239	2233	2236	2362	2274	2285	2187	2206	2587	2400	2410
After 100 F-T	Com. strength * (MPa)	43.7	46.8	51.4	60.5	50.1	53.9	41.3	46.8	50.2	46.8	50.1
	Flexural strength (MPa)	3.70	4.47	4.93	6.36	4.85	5.30	4.01	4.49	5.75	4.90	5.58
	Fracture energy (N/m)	225.2	362.4	482.2	1035.6	613.8	716.8	636.9	768.8	1691.8	816.1	995.6
	Max. def. ** (mm)	2.3	3.7	4.5	5.8	4.5	5.2	5.5	5.9	10.1	6.2	6.5
	DUW *** (kg/m^3^)	2041	2105	2123	2169	2118	2142	2042	2078	2286	2209	2235
After Acid	Com. strength * (MPa)	31.3	38.8	43.2	47.8	41	43.9	34.9	40.4	38	37.3	40.4
	Flexural strength (MPa)	2.91	3.62	4.08	5.01	3.88	4.24	3.38	3.93	4.56	3.77	4.36
	Fracture energy (N/m)	172.9	329.8	432	889.6	552.5	661.2	591.7	697.5	1243.4	782.9	968.2
	Max. def. ** (mm)	1.6	2.5	3	3.9	3.1	3.5	3.7	4	6.9	4.2	4.4
	DUW *** (kg/m^3^)	1810	1961	1988	1955	1956	1960	1911	1941	2049	2050	2073

* Compressive strength. ** Maximum deflection. *** Dry unit weight.

**Table 4:** CO_2_ emissions and unit costs of materials used in GM.

	BFS	NaOH	Na_2_SiO_3_	WMP	RFA	UTHF	THF	WTSF
CO_2_-e (kgCO_2_/kg)	0.0143 [111]	0.46 [15]	0.44 [112]	0.008 [113]	0.0018 [114]	−1.73 [115]	−0.85 [50]	0.58 [116]
Unit cost (USD/kg)	0.056	0.198	0.138	0.023	0.062	0.83	1.12	0.16

## Data Availability

The original contributions presented in this study are included in the article. Further inquiries can be directed to the author.

## References

[1] Aydin A.C., Karakoç M., Düzgün O., Bayraktutan M. (2010). Effect of low quality aggregates on the mechanical properties of lightweight concrete. Sci. Res. Essays.

[2] Jagadesh P., Nagarajan V. (2022). Effect of nano titanium di oxide on mechanical properties of fly ash and ground granulated blast furnace slag based geopolymer concrete. J. Build. Eng..

[3] Yang Z., Chen Z., Zhu H., Zhang B., Dong Z., Zhan X. (2024). Efficient utilization of coral waste for internal curing material to prepare eco-friendly marine geopolymer concrete. J. Environ. Manag..

[4] Guo Y., Luo L., Liu T., Hao L., Li Y., Liu P., Zhu T. (2024). A review of low-carbon technologies and projects for the global cement industry. J. Environ. Sci..

[5] Sousa V., Bogas J.A., Real S., Meireles I. (2023). Industrial production of recycled cement: Energy consumption and carbon dioxide emission estimation. Environ. Sci. Pollut. Res..

[6] Ding Y., Dai J.-G., Shi C.-J. (2016). Mechanical properties of alkali-activated concrete: A state-of-the-art review. Constr. Build. Mater..

[7] Davidovits J. The polysialate terminology: A very useful and simple model for the promotion and understanding of green-chemistry. Geopolymer chemistry and sustainable development solutions. Proceedings of the World Congress Geopolymer 2005.

[8] Ren D., Yan C., Duan P., Zhang Z., Li L., Yan Z. (2017). Durability performances of wollastonite, tremolite and basalt fiber-reinforced metakaolin geopolymer composites under sulfate and chloride attack. Constr. Build. Mater..

[9] She Y., Chen Y., Li L., Xue L., Yu Q. (2023). Understanding the generation and evolution of hydrophobicity of silane modified fly ash/slag based geopolymers. Cem. Concr. Compos..

[10] Yip C.K., Lukey G.C., Provis J.L., Van Deventer J.S. (2008). Effect of calcium silicate sources on geopolymerisation. Cem. Concr. Res..

[11] da Silveira Maranhão F., de Souza Junior F.G., Soares P., Alcan H.G., Çelebi O., Bayrak B., Kaplan G., Aydın A.C. (2023). Physico-mechanical and microstructural properties of waste geopolymer powder and lime-added semi-lightweight geopolymer concrete: Efficient machine learning models. J. Build. Eng..

[12] Kim Y.Y., Lee B.-J., Saraswathy V., Kwon S.-J. (2014). Strength and durability performance of alkali-activated rice husk ash geopolymer mortar. Sci. World J..

[13] Nassar A.K., Sivanandam S., Saran D., Sundar S.S., Kathirvel P., Murali G., Dixit S. (2025). Durability assessment of sustainable one-part alkali activated concrete produced from agricultural and industrial waste activators under aggressive environmental conditions. Constr. Build. Mater..

[14] Tchakouté H.K., Rüscher C.H., Kong S., Kamseu E., Leonelli C. (2017). Thermal behavior of metakaolin-based geopolymer cements using sodium waterglass from rice husk ash and waste glass as alternative activators. Waste Biomass Valorization.

[15] Zhang Y., Liu H., Ma T., Gu G., Chen C., Hu J. (2023). Understanding the changes in engineering behaviors and microstructure of FA-GBFS based geopolymer paste with addition of silica fume. J. Build. Eng..

[16] Marathe S., Sheshadri A., Nikolaiev V. (2025). Life cycle assessment of sustainable air-cured alkali-activated concrete for permeable pavements using agro-industrial wastes. Sci. Rep..

[17] Shi X., Zhang C., Liang Y., Luo J., Wang X., Feng Y., Li Y., Wang Q., Abomohra A.E.-F. (2021). Life cycle assessment and impact correlation analysis of fly ash geopolymer concrete. Materials.

[18] Turner L.K., Collins F.G. (2013). Carbon dioxide equivalent (CO_2−e_) emissions: A comparison between geopolymer and OPC cement concrete. Constr. Build. Mater..

[19] Odeh A., Al-Fakih A., Alghannam M., Al-Ainya M., Khalid H., Al-Shugaa M.A., Thomas B.S., Aswin M. (2024). Recent Progress in Geopolymer Concrete Technology: A Review. Iran. J. Sci. Technol. Trans. Civ. Eng..

[20] Shahmansouri A.A., Bengar H.A., Ghanbari S. (2020). Compressive strength prediction of eco-efficient GGBS-based geopolymer concrete using GEP method. J. Build. Eng..

[21] Zhao J., Tong L., Li B., Chen T., Wang C., Yang G., Zheng Y. (2021). Eco-friendly geopolymer materials: A review of performance improvement, potential application and sustainability assessment. J. Clean. Prod..

[22] Gao X., Yu Q., Yu R., Brouwers H. (2017). Evaluation of hybrid steel fiber reinforcement in high performance geopolymer composites. Mater. Struct..

[23] Murali G., Nassar A.K., Swaminathan M., Kathirvel P., Wong L.S. (2024). Effect of silica fume and glass powder for enhanced impact resistance in GGBFS-based ultra high-performance geopolymer fibrous concrete: An experimental and statistical analysis. Def. Technol..

[24] Ranjbar N., Zhang M. (2020). Fiber-reinforced geopolymer composites: A review. Cem. Concr. Compos..

[25] Farhan K.Z., Johari M.A.M., Demirboğa R. (2021). Impact of fiber reinforcements on properties of geopolymer composites: A review. J. Build. Eng..

[26] Lin T., Jia D., Wang M., He P., Liang D. (2009). Effects of fibre content on mechanical properties and fracture behaviour of short carbon fibre reinforced geopolymer matrix composites. Bull. Mater. Sci..

[27] Marathe S., Shetty Kuthyaru S., Bhat A.K. (2025). Stabilization of Indian Lateritic Subgrade Soil Using Alkali-Activated Slag with Sugarcane Bagasse Ash for Sustainable Pavement Infrastructure. J. Struct. Des. Constr. Pract..

[28] Shaikh F.U.A. (2013). Review of mechanical properties of short fibre reinforced geopolymer composites. Constr. Build. Mater..

[29] Korniejenko K., Lin W.-T., Šimonová H. (2020). Mechanical properties of short polymer fiber-reinforced geopolymer composites. J. Compos. Sci..

[30] Ma C.-K., Awang A.Z., Omar W. (2018). Structural and material performance of geopolymer concrete: A review. Constr. Build. Mater..

[31] Sakamoto K., Kawajiri K., Hatori H., Tahara K. (2022). Impact of the manufacturing processes of aromatic-polymer-based carbon fiber on life cycle greenhouse gas emissions. Sustainability.

[32] Silva G., Kim S., Aguilar R., Nakamatsu J. (2020). Natural fibers as reinforcement additives for geopolymers–A review of potential eco-friendly applications to the construction industry. Sustain. Mater. Technol..

[33] Ali H.A., Keke S., Xiaohao S., Poon C.S., Banthia N. (2025). Utilizing Incinerated Sewage Sludge Ash for Antibacterial Alkali-Activated Materials. Cem. Concr. Compos..

[34] Aydın A.C., Alcan H.G., Bayrak B., Kılıç M., Maali M. (2020). The mechanical behavior of thermally enhanced polypropylene concrete. Constr. Build. Mater..

[35] McNeil K., Kang T.H.-K. (2013). Recycled concrete aggregates: A review. Int. J. Concr. Struct. Mater..

[36] Merli R., Preziosi M., Acampora A., Lucchetti M.C., Petrucci E. (2020). Recycled fibers in reinforced concrete: A systematic literature review. J. Clean. Prod..

[37] Formela K. (2021). Sustainable development of waste tires recycling technologies–recent advances, challenges and future trends. Adv. Ind. Eng. Polym. Res..

[38] Zhao J., Xie J., Wu J., Zhao C., Zhang B. (2023). Workability, compressive strength, and microstructures of one-part rubberized geopolymer mortar. J. Build. Eng..

[39] Althoey F., Zaid O., Alsharari F., Yosri A.M., Isleem H.F. (2025). Evaluating the impact of nano-silica on characteristics of self-compacting geopolymer concrete with waste tire steel fiber. Arch. Civ. Mech. Eng..

[40] Mucsi G., Szenczi Á., Nagy S. (2018). Fiber reinforced geopolymer from synergetic utilization of fly ash and waste tire. J. Clean. Prod..

[41] Zhuo K.-X., Cai Y.-J., Lai H.-M., Chen Z.-B., Guo Y.-C., Chen G., Xiao S.-H., Lan X.-W. (2023). Axial compressive behavior of environmentally friendly high-strength concrete: Effects of recycled tire steel fiber and rubber powder. J. Build. Eng..

[42] Qin X., Kaewunruen S. (2022). Environment-friendly recycled steel fibre reinforced concrete. Constr. Build. Mater..

[43] Rehman M., Fahad S., Du G., Cheng X., Yang Y., Tang K., Liu L., Liu F.-H., Deng G. (2021). Evaluation of hemp (*Cannabis sativa* L.) as an industrial crop: A review. Environ. Sci. Pollut. Res..

[44] Nam T.H., Ogihara S., Tung N.H., Kobayashi S. (2011). Effect of alkali treatment on interfacial and mechanical properties of coir fiber reinforced poly (butylene succinate) biodegradable composites. Compos. Part B Eng..

[45] Shahzad A. (2012). Hemp fiber and its composites–A review. J. Compos. Mater..

[46] Arehart J.H., Nelson W.S., Srubar W.V. (2020). On the theoretical carbon storage and carbon sequestration potential of hempcrete. J. Clean. Prod..

[47] Zampori L., Dotelli G., Vernelli V. (2013). Life cycle assessment of hemp cultivation and use of hemp-based thermal insulator materials in buildings. Environ. Sci. Technol..

[48] Galzerano B., Formisano A., Durante M., Iucolano F., Caputo D., Liguori B. (2018). Hemp reinforcement in lightweight geopolymers. J. Compos. Mater..

[49] Lv C., Liu J. (2023). Alkaline degradation of plant fiber reinforcements in geopolymer: A review. Molecules.

[50] Shah I., Jing L., Fei Z.M., Yuan Y.S., Farooq M.U., Kanjana N. (2022). A review on chemical modification by using sodium hydroxide (NaOH) to investigate the mechanical properties of sisal, coir and hemp fiber reinforced concrete composites. J. Nat. Fibers.

[51] Qiu J., Zhao Y., Xing J., Sun X. (2019). Fly Ash/Blast Furnace Slag-Based Geopolymer as a Potential Binder for Mine Backfilling: Effect of Binder Type and Activator Concentration. Adv. Mater. Sci. Eng..

[52] Zhuguo L., Sha L. (2018). Carbonation resistance of fly ash and blast furnace slag based geopolymer concrete. Constr. Build. Mater..

[53] Aliabdo A.A., Abd Elmoaty M., Auda E.M. (2014). Re-use of waste marble dust in the production of cement and concrete. Constr. Build. Mater..

[54] Bayrak B., Benli A., Alcan H.G., Çelebi O., Kaplan G., Aydın A.C. (2023). Recycling of waste marble powder and waste colemanite in ternary-blended green geopolymer composites: Mechanical, durability and microstructural properties. J. Build. Eng..

[55] Petrović E.K., Thomas C.A. (2024). Global Patterns in Construction and Demolition Waste (C&DW) Research: A Bibliometric Analysis Using VOSviewer. Sustainability.

[56] Nematollahi B., Sanjayan J. (2014). Effect of different superplasticizers and activator combinations on workability and strength of fly ash based geopolymer. Mater. Des..

[57] Nodehi M., Taghvaee V.M. (2022). Alkali-activated materials and geopolymer: A review of common precursors and activators addressing circular economy. Circ. Econ. Sustain..

[58] John M.J., Anandjiwala R.D. (2008). Recent developments in chemical modification and characterization of natural fiber-reinforced composites. Polym. Compos..

[59] Alves C., Dias A., Diogo A., Ferrão P., Luz S., Silva A., Reis L., Freitas M. (2011). Eco-composite: The effects of the jute fiber treatments on the mechanical and environmental performance of the composite materials. J. Compos. Mater..

[60] Asokan R., Rathanasamy R., Paramasivam P., Chinnasamy M., Pal S.K., Palaniappan S.K. (2023). Hemp composite. Green Sustainable Process for Chemical and Environmental Engineering and Science.

[61] Mwaikambo L.Y., Ansell M.P. (2006). Mechanical properties of alkali treated plant fibres and their potential as reinforcement materials. I. hemp fibres. J. Mater. Sci..

[62] Sadeghi P., Cao Q., Abouzeid R., Shayan M., Koo M., Wu Q. (2024). Experimental and Statistical Investigations for Tensile Properties of Hemp Fibers. Fibers.

[63] Yan L., Kasal B., Huang L. (2016). A review of recent research on the use of cellulosic fibres, their fibre fabric reinforced cementitious, geo-polymer and polymer composites in civil engineering. Compos. Part B Eng..

[64] (2021). Standard Specification for Flow Table for Use in Tests of Hydraulic Cement.

[65] Alcan H.G., Bayrak B., Öz A., Kavaz E., Kaplan G., Çelebi O., Aydın A.C. (2023). A comprehensive characterization on geopolymer concretes with low content slag and quartz aggregates: The shielding features. Radiat. Eff. Defects Solids.

[66] Rovnaník P. (2010). Effect of curing temperature on the development of hard structure of metakaolin-based geopolymer. Constr. Build. Mater..

[67] (2022). Standard Test Method for Density, Absorption, and Voids in Hardened Concrete.

[68] (2018). Standard Test Method for Compressive Strength of Hydraulic-Cement Mortars (Using Portions of Prisms Broken in Flexure).

[69] (2021). Standard Test Method for Flexural Strength of Hydraulic-Cement Mortars.

[70] (2017). Standard Test Method for Flexural Toughness and First-Crack Strength of Fiber-Reinforced Concrete (Using Beam With Third-Point Loading).

[71] (2022). Standard Test Methods for Fire Tests of Building Construction and Materials.

[72] (2017). Standard Test Method for Resistance of Concrete to Rapid Freezing and Thawing.

[73] (2020). Standard Test Methods for Chemical Resistance of Mortars, Grouts, and Monolithic Surfacings and Polymer Concretes.

[74] Yazıcı Ş., İnan G., Tabak V. (2007). Effect of aspect ratio and volume fraction of steel fiber on the mechanical properties of SFRC. Constr. Build. Mater..

[75] Li Z., Wang L., Wang X. (2004). Compressive and flexural properties of hemp fiber reinforced concrete. Fibers Polym..

[76] Öz A., Bayrak B., Kaplan G., Aydın A.C. (2023). Effect of waste colemanite and PVA fibers on GBFS-Metakaolin based high early strength geopolymer composites (HESGC): Mechanical, microstructure and carbon footprint characteristics. Constr. Build. Mater..

[77] Madandoust R., Ranjbar M.M., Ghavidel R., Shahabi S.F. (2015). Assessment of factors influencing mechanical properties of steel fiber reinforced self-compacting concrete. Mater. Des..

[78] Xu Z., Zhang J., Zhang J., Deng Q., Xue Z., Huang G., Huang X. (2024). Influence of steel slag and steel fiber on the mechanical properties, durability, and life cycle assessment of ultra-high performance geopolymer concrete. Constr. Build. Mater..

[79] da Costa Santos A.C., Archbold P. (2022). Suitability of surface-treated flax and hemp fibers for concrete reinforcement. Fibers.

[80] Alsaif A.S., Albidah A.S. (2022). Compressive and flexural characteristics of geopolymer rubberized concrete reinforced with recycled tires steel fibers. Mater. Today Proc..

[81] Pham K.V., Nguyen T.K., Le T.A., Han S.W., Lee G., Lee K. (2019). Assessment of performance of fiber reinforced geopolymer composites by experiment and simulation analysis. Appl. Sci..

[82] Poletanovic B., Janotka I., Janek M., Bacuvcik M., Merta I. (2021). Influence of the NaOH-treated hemp fibres on the properties of fly-ash based alkali-activated mortars prior and after wet/dry cycles. Constr. Build. Mater..

[83] Ding Y., Bai Y.-L. (2018). Fracture properties and softening curves of steel fiber-reinforced slag-based geopolymer mortar and concrete. Materials.

[84] Gülşan M.E., Alzeebaree R., Rasheed A.A., Niş A., Kurtoğlu A.E. (2019). Development of fly ash/slag based self-compacting geopolymer concrete using nano-silica and steel fiber. Constr. Build. Mater..

[85] Suwan T., Maichin P., Fan M., Jitsangiam P., Tangchirapat W., Chindaprasirt P. (2022). Influence of alkalinity on self-treatment process of natural fiber and properties of its geopolymeric composites. Constr. Build. Mater..

[86] Bhutta A., Farooq M., Banthia N. (2019). Performance characteristics of micro fiber-reinforced geopolymer mortars for repair. Constr. Build. Mater..

[87] Amran M., Al-Fakih A., Chu S., Fediuk R., Haruna S., Azevedo A., Vatin N. (2021). Long-term durability properties of geopolymer concrete: An in-depth review. Case Stud. Constr. Mater..

[88] Manzoor T., Bhat J.A., Shah A.H. (2024). Performance of geopolymer concrete at elevated temperature−A critical review. Constr. Build. Mater..

[89] Grubeša I.N., Marković B., Gojević A., Brdarić J. (2018). Effect of hemp fibers on fire resistance of concrete. Constr. Build. Mater..

[90] Lakew A.M., Canpolat O., Al-Mashhadani M.M., Uysal M., Niş A., Aygörmez Y., Bayati M. (2023). Combined effect of using steel fibers and demolition waste aggregates on the performance of fly ash/slag based geopolymer concrete. Eur. J. Environ. Civ. Eng..

[91] Bayraktar O.Y., Tobbala D.E., Turkoglu M., Kaplan G., Tayeh B.A. (2023). Hemp fiber reinforced one-part alkali-activated composites with expanded perlite: Mechanical properties, microstructure analysis and high-temperature resistance. Constr. Build. Mater..

[92] Doğruyol M., Ayhan E., Karaşin A. (2024). Effect of waste steel fiber use on concrete behavior at high temperature. Case Stud. Constr. Mater..

[93] Min Y., Wu J., Li B., Zhang M., Zhang J. (2022). Experimental study of freeze–thaw resistance of a one-part geopolymer paste. Case Stud. Constr. Mater..

[94] Aygörmez Y., Canpolat O., Al-Mashhadani M.M., Uysal M. (2020). Elevated temperature, freezing-thawing and wetting-drying effects on polypropylene fiber reinforced metakaolin based geopolymer composites. Constr. Build. Mater..

[95] Farhan K.Z., Johari M.A.M., Demirboğa R. (2022). Evaluation of properties of steel fiber reinforced GGBFS-based geopolymer composites in aggressive environments. Constr. Build. Mater..

[96] Brooks R., Bahadory M., Tovia F., Rostami H. (2010). Properties of alkali-activated fly ash: High performance to lightweight. Int. J. Sustain. Eng..

[97] Fan L., Meng W., Teng L., Khayat K.H. (2019). Effect of steel fibers with galvanized coatings on corrosion of steel bars embedded in UHPC. Compos. Part B Eng..

[98] Syed M., GuhaRay A., Goel D., Asati K., Peng L. (2020). Effect of freeze–thaw cycles on black cotton soil reinforced with coir and hemp fibres in alkali-activated binder. Int. J. Geosynth. Ground Eng..

[99] Ghosn S., Cherkawi N., Hamad B. (2020). Studies on hemp and recycled aggregate concrete. Int. J. Concr. Struct. Mater..

[100] Mohebi R., Behfarnia K., Shojaei M. (2015). Abrasion resistance of alkali-activated slag concrete designed by Taguchi method. Constr. Build. Mater..

[101] Wang J., Niu D. (2016). Influence of freeze–thaw cycles and sulfate corrosion resistance on shotcrete with and without steel fiber. Constr. Build. Mater..

[102] Zhu F., Ma Z., Zhao T. (2016). Influence of Freeze-Thaw Damage on the Steel Corrosion and Bond-Slip Behavior in the Reinforced Concrete. Adv. Mater. Sci. Eng..

[103] Pradhan P., Dwibedy S., Pradhan M., Panda S., Panigrahi S.K. (2022). Durability characteristics of geopolymer concrete-Progress and perspectives. J. Build. Eng..

[104] Liu H., You L., Jin H., Yu W. (2013). Influence of alkali treatment on the structure and properties of hemp fibers. Fibers Polym..

[105] Saranya P., Nagarajan P., Shashikala A.P. (2021). Performance studies on steel fiber–Reinforced GGBS-dolomite geopolymer concrete. J. Mater. Civ. Eng..

[106] Thanushan K., Sathiparan N. (2022). Mechanical performance and durability of banana fibre and coconut coir reinforced cement stabilized soil blocks. Materialia.

[107] Rabie M., Irshidat M.R., Al-Nuaimi N. (2022). Ambient and heat-cured geopolymer composites: Mix design optimization and life cycle assessment. Sustainability.

[108] Salas D.A., Ramirez A.D., Ulloa N., Baykara H., Boero A.J. (2018). Life cycle assessment of geopolymer concrete. Constr. Build. Mater..

[109] Gu X., Li Z., Zhang Y., Zhang W., Li X., Liu B. (2024). Sustainable assessment and synergism of ceramic powder and steel slag in iron ore tailings-based concrete. Environ. Sci. Pollut. Res..

[110] Yakhlaf M., Safiuddin M., Soudki K. (2013). Properties of freshly mixed carbon fibre reinforced self-consolidating concrete. Constr. Build. Mater..

[111] García-Segura T., Yepes V., Alcalá J. (2014). Life cycle greenhouse gas emissions of blended cement concrete including carbonation and durability. Int. J. Life Cycle Assess..

[112] Davidovits J. (2015). False values on CO2 emission for geopolymer cement/concrete published in scientific papers. Tech. Pap..

[113] Sánchez A.R., Ramos V.C., Polo M.S., Ramón M.V.L., Utrilla J.R. (2021). Life cycle assessment of cement production with marble waste sludges. Int. J. Environ. Res. Public Health.

[114] Bampanis I., Vasilatos C. (2023). Recycling Concrete to Aggregates. Implications on CO_2_ Footprint. Mater. Proc..

[115] Di Sarno L., Albuhairi D., Medeiros J.M.P. (2024). Exploring innovative resilient and sustainable bio-materials for structural applications: Hemp-fibre concrete. Structures.

[116] Wang Y., Qiao P., Sun J., Li H., Chen A. (2025). Production of ultra-high performance concrete using recycled tire steel fiber: Mechanical properties and life cycle assessment. Mag. Concr. Res..

[117] Abushanab A., Alnahhal W. (2023). Life cycle cost analysis of sustainable reinforced concrete buildings with treated wastewater, recycled concrete aggregates, and fly ash. Results Eng..

[118] Alcan H.G., Bayrak B., Öz A., Çelebi O., Kaplan G., Aydın A.C. (2024). Synergetic effect of fibers on geopolymers: Cost-effective and sustainable perspective. Constr. Build. Mater..

[119] Nikbin I.M., Dezhampanah S., Charkhtab S., Mehdipour S., Shahvareh I., Ebrahimi M., Pournasir A., Pourghorban H. (2022). Life cycle assessment and mechanical properties of high strength steel fiber reinforced concrete containing waste PET bottle. Constr. Build. Mater..

